# Action design research to develop an interactive dashboard to visualise and compare patient data from Irish general practice (CARA)

**DOI:** 10.1136/bmjopen-2024-086677

**Published:** 2025-09-04

**Authors:** Heike Vornhagen, Nathaly Garzón-Orjuela, Katarzyna Stasiewicz, Agustin Garcia Pereira, Sana Parveen, Lukasz Porwol, Claire Collins, Catherine Blake, Akke Vellinga

**Affiliations:** 1 Insight Centre for Data Analytics, University of Galway, Galway, Ireland; 2 School of Public Health, Physiotherapy and Sports Science, University College Dublin, Dublin, Ireland; 3 CARA Network, School of Public Health, Physiotherapy and Sports Science, University College Dublin, Dubln, Ireland; 4 Research Centre, Irish College of General Practitioners, Dublin, Ireland; 5 Public Health and Primary Care, Ghent University, Gent, Belgium

**Keywords:** Primary Health Care, Clinical audit, Health Services

## Abstract

**Abstract:**

**Objective:**

A dashboard was developed with and for Irish general practitioners (GPs) to improve their understanding of practice data. The aim of this study was to design and develop interactive CARA dashboards to enable Irish GPs to visualise patient data and compare their data with other practices.

**Design:**

An interpretivist qualitative approach was taken to create a deeper understanding of how GPs view and engage with data. It included four stages: (a) problem formulation, (b) building, intervention and evaluation, (c) reflection and learning and (d) formalisation of learning. The process included interviews to explore what type of information GPs need, as well as iterative testing of the CARA dashboard prototype.

**Setting:**

General practice.

**Participants:**

GPs, design experts and domain experts (antibiotic prescribing and stewardship).

**Results:**

Key challenges identified from the interviews (context, sense-making, audits, relevance, action, engagement and ease of use) formed the basis for developing the CARA dashboard prototype. The first exemplar dashboard focused on antibiotic prescribing to develop and showcase the proposed platform, including automated audit reports, filters (within-practice) and between-practice comparisons, as well as a visual overview of practice demographics. The design thinking approach helped to capture and build an understanding of the GPs’ perspectives and identify unmet needs. This approach benefits the quality improvement methodology commonly adopted across healthcare, which aims to understand the process, not the users.

**Conclusions:**

The development of a *useful* dashboard is based on two key elements: users’ requirements and their continued involvement in the development of content and overall design decisions. The next step will be an incremental inclusion of GPs using the dashboard and an exploratory study on dashboard engagement. Additional dashboards, such as for chronic disease, will be developed.

**STRENGTHS AND LIMITATIONS OF THIS STUDY**

- User involvement was incorporated throughout the dashboard design process to align with engagement and usability needs.
- The study applied the Action Design Research framework to guide iterative development and evaluation of the dashboard.
- The problem formulation stage was informed by qualitative interviews with GPs, grounding the design in real-world context.
- The limited number of interviews may not fully represent the diversity of the user population (GPs).
- The CARA project has yet to be fully implemented in real-world settings, limiting the evaluation of its impact on prescribing behaviour and long-term adoption.

## Introduction

 Health informatics technology has been transforming health and healthcare delivery programmes worldwide. While information visualisation and visual analytical processes are central to fully achieving this transformation, their application in health is considerably less commonplace compared with other scientific disciplines.^1^ Challenges arise across many areas, including data integration, scope of use and data complexity, which includes heterogeneous variables, multiple sources, missing and incomplete data and statistical rigour.^1 2^ However, healthcare encounters additional barriers due to stringent privacy regulations, the high cost of clinical errors and the necessity for interpretations to align with established medical protocols.^3 4^ Unlike other fields where exploratory analysis is encouraged, healthcare decisions require high precision, limiting the flexibility of visual analytics and thereby slowing their adoption.^3 4^

Visualisation of information through digital (or interactive) dashboards is growing in the health sector, in particular since the pandemic, and has applications in patient management and pharmacological prescribing practice. However, dashboards are typically launched for a single purpose, such as research, exploration, analysis or business. Most dashboards are designed ‘for’ users rather than ‘with’ them, offering limited or no user interaction or engagement.^5^,^7^ Once set up, dashboards require ongoing input from the developers to maintain their relevance,^5^ which must align with the end users’ needs. Developers should apply iterative performance evaluations during the development and implementation phase to encourage uptake and use.^8^ Researchers and developers must ensure the dashboard remains effective and relevant in terms of both health outcomes and user engagement. Dashboard users require context and transparency in data transformation processes, while adherence to design principles enhances data interpretation.^9 10^ It is important to involve users when designing visualisation tools or computerised audit and feedback systems in healthcare.^11 12^

The use of health data dashboards surged with the public health response to the pandemic, which demonstrated the need and value of timely, publicly available data.^13^ For instance, the WHO and the European Centre for Disease Prevention and Control developed dashboards to visualise COVID-19 data collected by countries.^14 15^ The COVID-19 pandemic also exposed the lack of data standards and the problem of data silos, where data are stored in isolated systems.^13^ Visualising COVID-19 data helped the pandemic response but did not solve the data silo issues. A similar situation is often observed in primary and secondary care, where data integration and exchange are haphazard.^16^

The Irish health system is characterised by a complex mix of public and private financing and services delivery.^17^ General practice is the foundation of primary healthcare, critical within the Irish health services, with approximately 1635 practices and nearly 3500 active general practitioners (GPs).^18^ However, Irish GPs differ from those in many other countries, since they operate independently as private professionals within general practices that serve as private businesses.^16^ Nearly all general practices are computerised and the practice is the legal data controller of the patient data, which is stored in a practice database.^18^

Irish GPs have patient management software (PMS) systems to conduct patient consultations and manage patient medical records.^19^ PMS is organised around the consultation, making it difficult for GPs to access data for audits that require an aggregated understanding of their patients. A clinical audit is a yearly requirement as part of the GP’s clinical competence certification by the Medical Council, and research has shown that such audits increase participation and commitment from GPs.^19 20^ The government has implemented some initiatives, such as Summary Care Records and ePrescribing, which aim to improve interoperability. However, these efforts have not resulted in the integration of data visualisation tools for general practice.^21^ Moreover, data integration and visualisation remain underdeveloped in key areas, particularly in prescribing feedback mechanisms. The Health Service Executive Primary Care Reimbursement Service has introduced quarterly antibiotic prescribing feedback for patients with a medical card.^22 23^ However, this feedback, which excludes information on private patients, is in PDF format and has been reported as complex, lacking interactive features.^24^ Although these initiatives aim to integrate health services, they are designed for specific purposes and rarely seek input from GPs during development or implementation. No initiative facilitates comparisons with other practices or national targets, making it difficult to benchmark and assess GPs’ prescribing and care delivery.

CARA is a funded research programme that set out to develop a data-sharing platform to facilitate GPs to develop a deeper understanding of their patient population, disease management and prescribing through dashboards. The first exemplar dashboards focused on antibiotic prescribing and practice overview to develop and showcase the proposed infrastructure, including automated audit reports, filters (within the practice) and between-practice comparisons, as well as an overview of practice demographics.^25^ Internationally, similar dashboards focused on antibiotic prescribing that include user involvement have been developed in Canada and the UK.^26 27^ However, these dashboards do not describe the process of user involvement in the system design, development and testing or use a methodological framework for developing the dashboard. A recent scoping review of dashboards in healthcare identified major shortcomings in how dashboards are typically developed.^28^ Of the 118 dashboards reviewed, only 50% reported involving end users in the development process, and even fewer (22%) included in usability testing.^28^ Informed by these shortcomings, as well as the fragmented data environment and limited visualisation tools with general practice data, CARA adopted a structured methodological framework with continuous GP participation, co-design principles and iterative testing. The aim of this study was to design and develop interactive CARA dashboards to enable Irish GPs to visualise their patient data and compare it with other practices.

## Methods

### Qualitative approach

**Figure 1:**
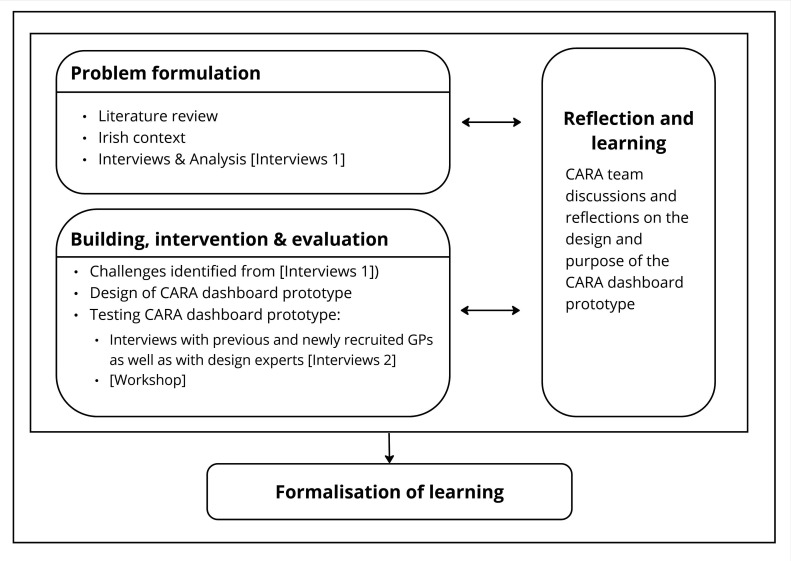
Overview of the ADR process for developing the CARA dashboard. Sources: adapted by authors based on the ADR from Sein *et al*.^30^ ADR, Action Design Research and GP, General Practitioner.

An interpretivist approach was taken to create a deeper understanding of how GPs view and engage with data. Action Design Research (ADR) generates knowledge about how to solve organisational problems in practice and integrates elements from Design Science Research and Action Research.^29 30^ The ADR process was structured in accordance with the four stages characterised by Sein *et al* (figure 1).^30^

ADR is an iterative process:

- *Problem formulation*: the starting point of the process involved defining and conceptualising the problem through a review of the literature. In addition, interviews were conducted with GPs and researchers to define the type of information that could be considered for visualisation in a dashboard (Interviews 1).
- *Building, intervention and evaluation*: a dashboard prototype was designed using the collaborative interface design tool Figma (http://www.figma.com/). The prototype was iteratively tested in a second round of interviews (Interviews 2), which included previous and newly recruited GPs and design experts. An adapted prototype was presented to GPs during an interactive workshop (at a national GP conference).
- *Reflection and learning:* this stage, alongside the first two stages, involved continuous reflection and discussion about the design and purpose of the dashboard prototype to solve specific issues.
- *Formalisation of learning:* the last stage involves three levels to formalise learning (generalisation of the problem instance, the solution instance and the derivation of a design principle).^30^

The primary outcome of this ADR process was the development of a prototype CARA dashboard tailored to Irish GPs, enabling visualisation of their data, comparison with other practices and audit generation. A key feature of this work is its iterative, co-designed development, informed directly by end-users through interviews and workshop feedback. The ADR approach also facilitated the generation of transferable design principles for dashboard development in general practice, representing a novel contribution to both dashboard design methodology and primary care informatics.

### Recruitment and sampling

Participants for two rounds of qualitative interviews were recruited through snowball sampling.^31^ Initial contact with participants was through personal communication. Participants were asked to identify other colleagues to participate. The inclusion criteria for the first set of interviews (Interviews 1) were GPs registered with the Irish Medical Council. The second set of interviews (Interviews 2) included the initial GPs interviewees (Interviews 1) plus newly recruited GPs and design or antimicrobial stewardship experts.

The CARA workshop was conducted during the national GP conference and participants were recruited from GPs registered for this event. This workshop involved a demonstration of the CARA dashboard followed by a practical session for the participating GPs to explore how the dashboard can support their data and audit needs.

### Data collection

Data collection for the two interview rounds was completed remotely on Zoom. All interviews were video recorded with consent, transcribed verbatim and identifiable data were removed. The transcripts were shared with the respective participants before analysis.

Interviews 1 were undertaken in April and May 2022 and focused on ‘*Problem formulation’,* using questions regarding information needs (eg, ‘What type of information would best support your reporting requirements?’) and user requirements (eg, ‘What would encourage you to visit and use a dashboard?’). Interviews 2 took place from October to November 2022, focusing on ‘*Building, intervention and evaluation’*. This user evaluation of the CARA prototype dashboard was divided into two phases:

- Completing a task while thinking out loud (cooperative evaluation): a task was presented to a GP (carry out an audit to reduce the overall number of antibiotics and, specifically, to reduce the prescription of red antibiotics).^32^ The GP was asked to verbalise their thoughts, and the interviewer used prompting questions such as ‘Why did you do that?’ and ‘What were you expecting to happen?’
- General evaluative discussion focusing on questions such as ‘What did you find useful/not useful?’, ‘What would you change?’ and ‘Can you see yourself using the dashboard (frequently/rarely/only for audits/not at all)?’

Data were collected from the CARA workshop (4 March 2023) through a questionnaire (open-ended and closed-ended questions; see online supplemental material 1).

### Data analysis

Analysis was conducted in three distinct phases, interviews were transcribed and coded using reflexive thematic analysis.^33^

- Interviews 1 consisted of an iterative analysis of the transcripts, identifying relevant phrases and coding these based on the research questions using Excel. Codes were grouped to define common themes. This analysis was carried out by a researcher (HV).
- In Interviews 2, the transcripts were coded using NVivo V.12, and analyses focused on different dashboard sections. Themes and categories were generated based on the task and discussion. These categorisations were carried out by a researcher (NG-O) and checked by a second researcher (HV).
- Each closed-ended question of the workshop’s questionnaire was synthesised in frequencies and percentages, and open-ended questions were aggregated into common categories for analysis.

### Patient and public involvement

Patients and/or the public were not involved in the design, conduct, reporting or dissemination plans of this research.

## Results

16 interviews were conducted in total: 6 in Interviews 1 with GPs (‘*Problem formulation*’) and 10 in Interviews 2, which included five GPs who had already participated in Interviews 1, two newly recruited GPs and three experts (‘*Building, intervention and evaluation*’).

### Problem formulation

The literature review showed that only two antibiotic dashboards were found to describe a development process that integrated users’ input.^6 26 27^ However, no evidence was found for any of the available dashboards to have included crucial components required to make sense of the information provided on these dashboards (such as the issues of context, transparency of data transformation processes, adherence to design principles and users’ ability to understand the data displayed).^6^ Given this gap in knowledge, six interviews (GPs) (Interviews 1) were conducted to identify common challenges that GPs, the key users, face when looking at their patient data. Once the initial coding was completed, the codes were reviewed and grouped to establish overarching themes (see online supplemental table 1). The key challenges identified were:

1. Context: dashboard design needs to adjust dynamically to the user’s context to acknowledge that GP practices operate in different contexts, with respect to size, location, age profiles, etc.
2. Sense-making: information presented must be understandable. Given the potential volume of information available, information presentation needs to be focused and clear.
3. Audits: support to carry out mandatory audits is crucial. Annual audits are a requirement for all GPs, and easy generation of audit reports can save GPs time, thereby offering an additional bonus.
4. Relevance: the dashboard must be relevant to its users. Data presented on the dashboard must be timely and relevant to the local context.
5. Action: the dashboard needs to have a clear message and support/encourage users to take action. The provision of clear messages about national prescribing targets and goals for improvement in practice should be used to motivate change.
6. Engagement: users’ motivations and ongoing engagement need to be supported to be useful; therefore, users need to be kept in the loop.
7. Ease of use: using a dashboard needs to be easy and efficient, as well as cater for all levels of users to allow both data novices and experts to interact with the information.

### Building, intervention and evaluation

**Figure 2:**
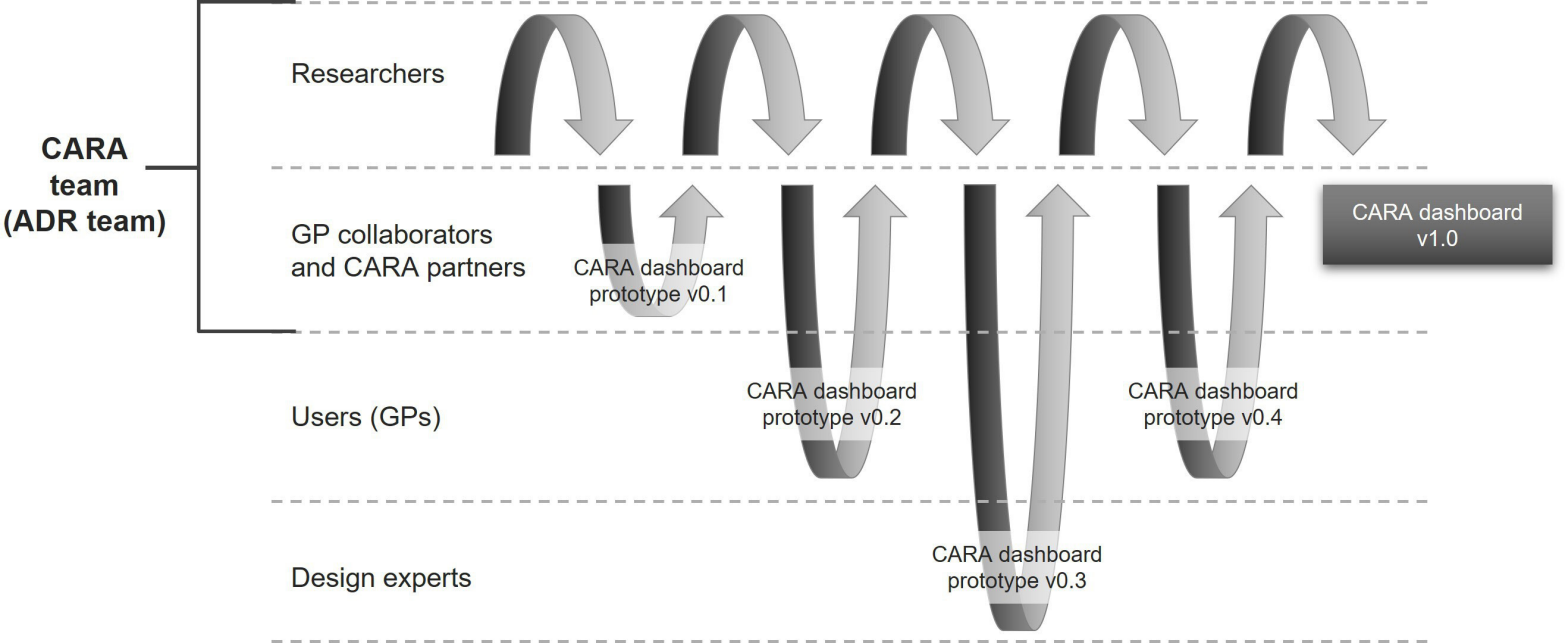
The design cycle of the CARA dashboard. Sources: adapted by authors based on the ADR from Sein *et al*.^30^ ADR, Action Design Research and GP, general practitioner.

Based on the key challenges identified in ‘*Problem formulation*’, the CARA dashboard prototype V.0.1 was developed for testing.^34^ The design cycle process from the CARA dashboard prototype V.0.1^34^,^37^ to the CARA dashboard V.1.0 (which was created specifically for the workshop and is no longer available; instead, a new DEMO version with synthetic practice data was created) is shown in figure 2.^38^ The ways in which specific challenges were addressed during the design process of the CARA dashboards are summarised in table 1. Further visual evidence (screenshots) from the design process of the CARA dashboard is provided in online supplemental table 2.

**Table 1:** Summary of how specific challenges were identified and addressed during the CARA dashboard design process

Challenge	Reasons	Addressed by	Prototype changes
Context	Currently, PMS used by GPs in Ireland do not allow practices to view overall patient data (eg, numbers, breakdowns of gender, age, etc). This information is useful, particularly for comparisons with other practices to facilitate benchmarking.	Practice data were restructured, and filters were calculated for age, gender, GMS/private and dates. Practice data were made visible through simple charts. When signing up, practices indicate area, location and staffing (FTE), thereby allowing practices to be grouped into categories (eg, rural practices, practices with 2 GPs (FTE), etc).	Filters were introduced in V.0.1,^34^ simplified in V.0.3^36^ and streamlined in V.0.4^37^ to improve responsiveness and overall look and feel. Charts were redesigned based on feedback throughout the prototype iterations.
Sense-making	As GPs can only access individual patient data through their PMS, it is challenging and time-consuming to view, for example, data on prescribing over time. Equally, while some feedback is provided through audits, audits are practice-specific and do not allow peer comparisons.	Categorisation of prescribing data and presentation of high-level overviews that can be adjusted through filters. Detailed information on antibiotic prescribing, which again can be filtered. Providing peer comparisons by showing averages of all participating practices.	A chart on prescriptions was added in V.0.3^36^ and refined in V.0.4.^37^ Charts on antibiotic prescribing were simplified between V.0.2^35^ and V.0.3.^36^ The chart on antibiotic prescriptions for specific diseases was removed in V.0.3,^36^ as it became clear that data were not available.
Audits	Audits are a yearly requirement for each GP in Ireland. At present, GPs have to identify patients’ data for download and manual aggregation. This process is prone to errors and is time-consuming.	Three simple audit goals for antibiotic prescribing were identified and transformed into a clickable audit report, which can be done in less than 5 min.	The process to carry out an audit was simplified between V.0.2^35^ and V.0.3,^36^ resulting in simpler navigation (fewer clicks) and improved layout.
Relevance	To keep dashboards relevant requires up-to-date data and includes all necessary variables to satisfy GPs’ information needs.	CARAconnect^25^ allows the GP to automate an upload of their practice data in a few simple steps.	Included notification of last data upload (V.0.1)^34^ and action button in demo. Charts were iteratively reviewed to ensure relevance to GPs.
Action	Highlighting the value of ICPC coding motivates GPs to implement it in their data recording. Showing how prescribing aligns with or deviates from guidelines supports informed, guideline-based decision-making.	Included key box summaries on the landing page to highlight priority insights at a glance and added charts showing ICPC codes by consultations and prescriptions to demonstrate their practical value. Antibiotic prescribing was translated to the green/red classification system to guide antibiotic prescribing in general practice.	Key box summaries, disease classification (ICPC code) charts by consultations and prescriptions, and the green/red antibiotic classification system were implemented in V.0.1,^34^ simplified in V.0.3,^36^ and streamlined in V.0.4.^37^ A top 10 consultations for chronic diseases (ICPC) was implemented in V.0.4.^37^
Engagement	Dashboards are only useful if users stay engaged. Engagement increases when the topic is directly relevant to daily practice and provides ongoing value. Regular, well-timed prompts help maintain interaction, while sustained engagement requires both user interest and available time. The ability to track progress over time provides additional motivation by showing trends and changes that inform decision-making.	Introduced a *Practice Overview* dashboard to provide a concise, high-level summary of practice data. Added *Key Summary* boxes and a green/red guideline system to highlight priority prescribing insights. Initially, the *Audit* was a separate dashboard, but based on GP feedback, the Audit was integrated into the *Antibiotics* dashboard. Replaced national averages with regional comparisons to improve peer benchmarking.	Practice Overview dashboard introduced in V.0.2^35^ and refined in V.0.3^36^ and V.0.4.^37^ Audit dashboard created in V.0.2^35^ and moved into Antibiotics dashboard in V.0.3.^36^ Key Summary boxes and green/red guideline system implemented in V.0.2.^35^ Regional average comparisons replaced national averages in V.0.3^36^ and were further improved in V.0.4.^37^
Ease of use	GPs have different digital skills and any data analysis platform needs to be intuitive and easy to use.	Use of simple charts (bar charts, line graphs and pie charts). Feedback throughout prototyping to address potentially confusing navigation or content.	Prototype V.0.1^34^ included bar charts, line graphs and pie charts, but also tree maps, butterfly charts and box plots. The latter were removed in subsequent iterations, as they caused confusion. Navigation was streamlined continuously with the final version V.0.4^37^ featuring main navigation on the left and filters on top.

FTE, Full-Time Equivalent; GMS, General Medical Services; GP, General Practitioner; ICPC, International Classification of Primary Care; PMS, Patient Management Software.

**Figure 3:**
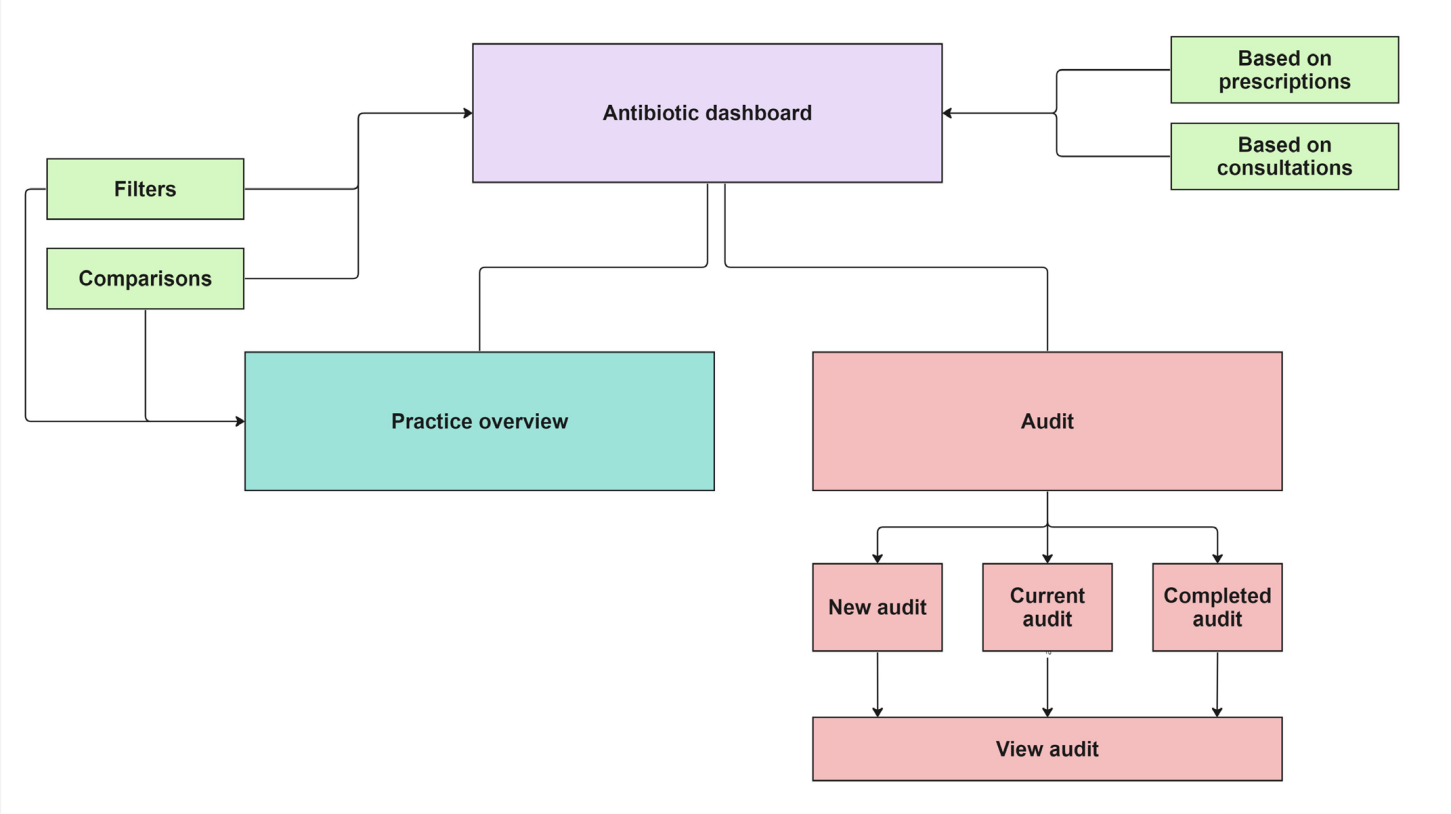
The main sections of the CARA dashboard prototype V.0.1.^34^

The prototype consisted of three main sections: the main antibiotics dashboard (with filters for both practice and patient characteristics), an audit section (including a new audit walkthrough and a mock audit report) and a practice overview (figure 3).^34^ The main antibiotics dashboard contained visualisations on antibiotics use and resistance, use of antibiotics over time and comparisons to the national average, thereby addressing challenges 2, 4, 5 and 7. Filters and in-between comparisons allowed users to adjust the visualisations according to their needs (challenges 1 and 4). The practice overview was seen as very useful by testers and would encourage them to access the dashboard frequently (challenge 6). The audit page directly addressed challenge 3 and included a list of goals and visualisations for users to choose for their analysis.

During the prototype testing (Interviews 2), 10 interviews were carried out, involving five GPs who had already participated in ‘*Problem formulation’* (Interviews 1), two newly recruited GPs, two design experts and an antibiotic stewardship expert. The interview codes were combined into eight themes, which emerged during the task and discussion. The main results of these themes and emerging categories (see online supplemental table 3) were discussed in ‘*Reflection and learning*’.

**Figure 4:**
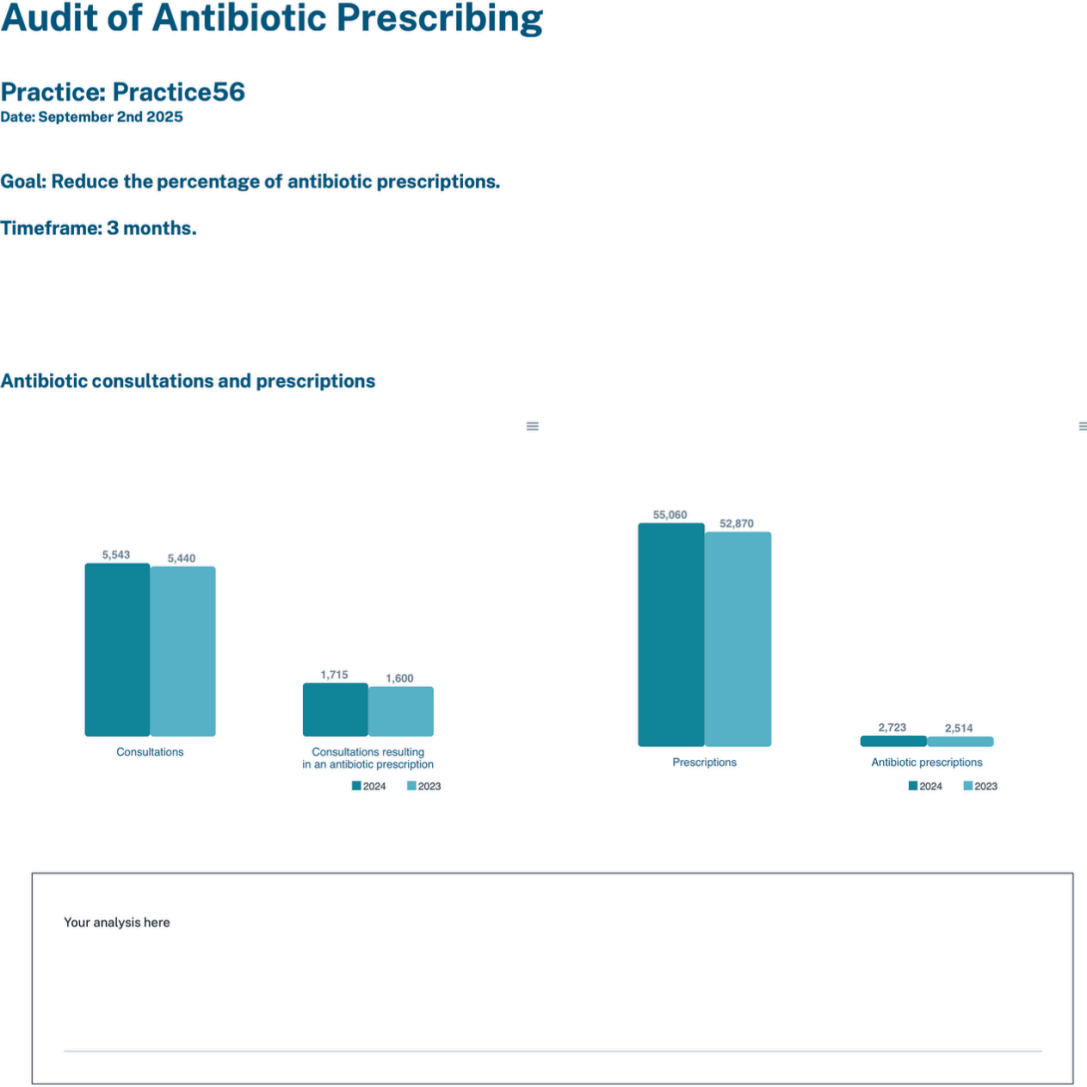
Sample of an audit report, CARA dashboard V.1.0.^38^

The prototype was iteratively adjusted to address concerns raised by the testers, resulting in a final prototype (CARA dashboard V.1.0).^38^ Changes were made to the audit page in the CARA dashboard V.1.0 to include a list of goals and timeframes for users to choose from and simplify coding requirements. GPs could choose to ‘reduce the percentage of antibiotic prescriptions’ over the next 3 months and would be presented with a visualisation appropriate to their selected goal (in this example, a bar chart showing the percentage of antibiotic prescriptions compared with all prescriptions/consultations for the 3 months and the same timeframe of the previous year). A textbox allows GPs to input their own analysis, and the report could then be downloaded and submitted or shared with colleagues (figure 4).

The CARA dashboard V.1.0 was next presented in an interactive workshop with 13 GPs. 11 GPs (84%) found navigating the dashboard easy and would use this platform one or two times per month. Two GPs indicated using it weekly. Six GPs suggested that it would be helpful to identify or filter by the individual prescriber (including GPs and general practice nurses). For the next step of the CARA dashboard, GPs would like to see visualisations on chronic disease (41%), vaccination (18%), opioid prescribing (18%) and mental health (14%).

### Reflection and learning

**Figure 5:**
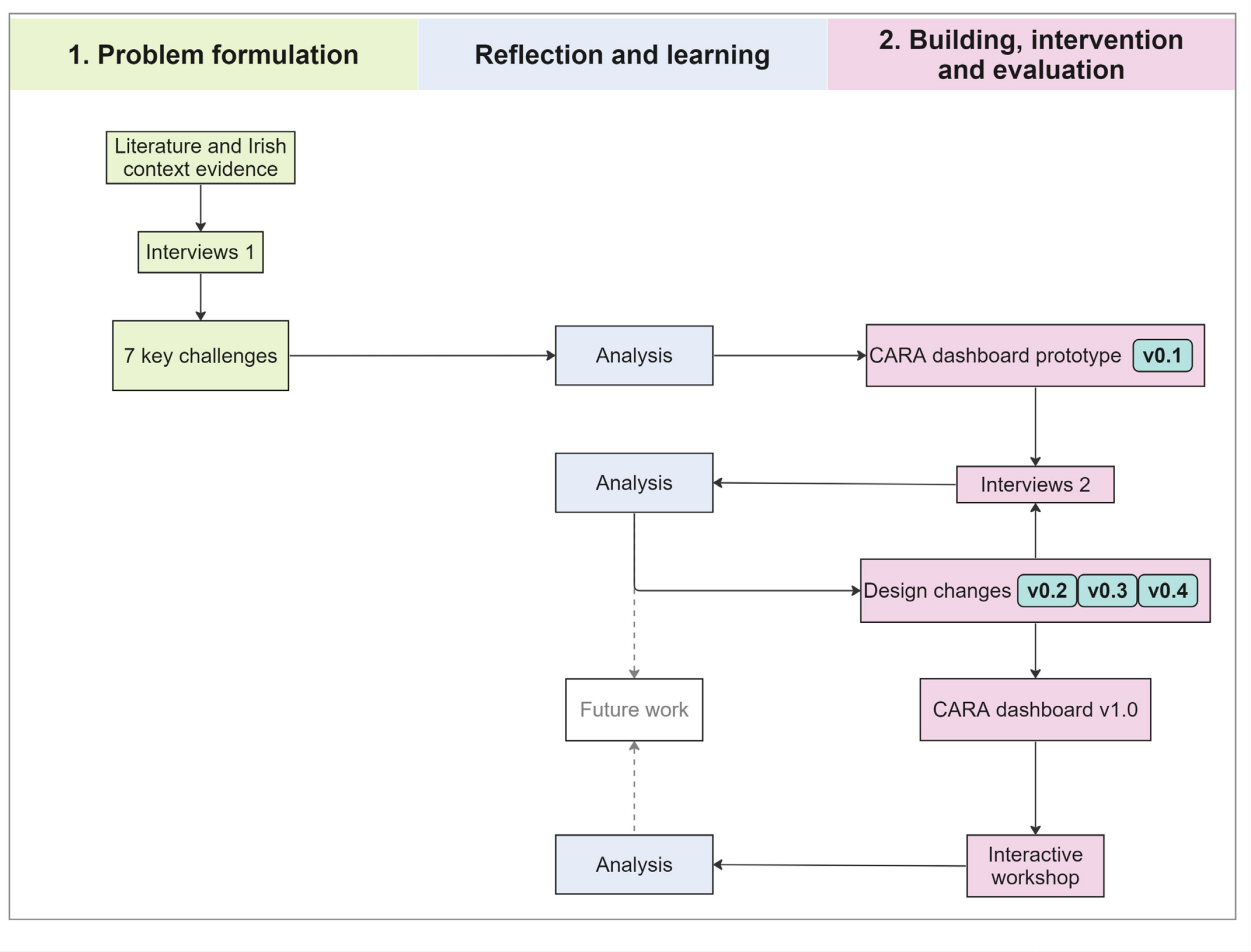
Action plan of reflection and learning in the CARA team.

This stage involved continuous reflection and discussion of specific points or issues from ‘*Problem formulation*’ and ‘*Building, intervention and evaluation*’ (figure 5).

The discussion points from ‘*Problem formulation*’ informed the first CARA dashboard prototype (V.0.1).^34^ Concurrent testing cycles were performed to review and adapt the prototype (‘*Building, intervention and evaluation*’). While some points emerging from the user evaluation (Interviews 2) directly informed the various CARA dashboard prototype iterations (design changes), other actions require longer-term implementation (future work). For instance, GPs reflected on the importance of including quality improvements by comparing their performance versus the gold standard or national target:

An audit is a process. So it’s about finding out the sort of standards of the target, changing something, and then re-checking’ (GP).

Furthermore, GPs suggested including other dashboards, such as for chronic diseases, of which the management includes remuneration per patient, to make this platform more attractive and increase engagement:

You don't get the remuneration for anything to do with antimicrobial prescribing or stewardship, so like the chronic disease management stuff … if you had that here, people would probably be using it all the time from a practice perspective (GP).

### Formalisation of learning

The problem identified and generalisable across GP practices was a lack of support to access patient data easily and meaningfully. Data silos, lack of data standards and an overall disregard for the real-life context of general practice make it harder for GPs to monitor and improve their prescribing. CARA developed both an infrastructure and, with GPs, co-designed a dashboard to overcome these challenges.

## Discussion

This paper describes the design and development of an interactive dashboard that enables GPs to visualise their patient data and compare it with other practices. Access to and understanding of patient data are essential for GPs to monitor and improve their medical practice. Currently, there are no easy-to-use tools available that support Irish GPs to achieve these goals. GPs’ views and experiences were used to inform and test prototype dashboards while ensuring their needs were iteratively addressed. The key elements were derived from user interviews and included the need to adjust to differing contexts, support for audits, relevance of data and support for user engagement.

Several challenges involving the implementation of healthcare visualisation tools and the processing and rendering of complex healthcare data sets have previously been identified.^1 39^ The CARA dashboards’ design addresses some of these challenges through (1) a visual analytics tool to allow GPs to view their data in the context of their practice; (2) providing access to and visualisation of the practice data, which is a step towards a new ecosystem of visual tools; (3) the co-design approach involving GPs to develop the CARA dashboard; (4) supporting decision making; (5) facilitating between-practice comparisons and (6) offering easy-to-generate audit reports.^1 39^

This study followed an ADR approach, which emphasises iterative problem-solving through cycles of artefact construction, intervention and evaluation.^30^ 16 interviews were conducted to inform the iterative process; however, the goal was not to achieve data saturation, as would be expected in traditional qualitative methodologies such as grounded theory. Instead, the aim was to support the ongoing refinement of the dashboard through continuous stakeholder engagement. Accordingly, the sample size was determined by the pragmatic requirements of the design process rather than by the pursuit of thematic saturation. Given the study’s interpretivist orientation, generalisability is analytical rather than statistical. Case-based and design-oriented research can yield theoretical insights that are transferable beyond the immediate empirical context.^40^ In this case, the lessons learnt about co-designing and implementing dashboards in general practice may have relevance for similar initiatives in other healthcare settings.

Only two existing dashboards on antibiotic prescribing that include user involvement have been developed in Canada and the UK.^26 27^ However, these do not describe the users’ involvement process in the system’s design, development and testing, nor do they use a methodological framework for developing the dashboard. Using the ADR process enabled an iterative reviewing and adapting cycle to incorporate feedback from GPs (bottom-up approach). Many dashboards are designed based on a desire to transfer information (data) to users without involving them in their design. The design thinking approach helped to capture and build an understanding of the GPs’ perspectives and identify unmet needs.^41 42^ This approach benefits the quality improvement methodology commonly adopted across healthcare, which aims to understand the process, not the users.^41^

The first exemplar CARA dashboard focused on antibiotic prescribing, as antibiotic resistance is an ongoing global health concern.^43^ However, in GP practices, patients present with a variety of health issues. Hence, the CARA team aims to explore and implement new dashboards, such as for chronic diseases, which will also contribute to the sustainability and engagement of the infrastructure. Continued updates and additions to the CARA infrastructure by expanding through new dashboards will ensure its longevity. This also sets CARA apart from other projects, as similar dashboards are designed for a single purpose (eg, antibiotic prescribing or diabetes), while CARA will use the antibiotics exemplar to develop and implement other dashboards based on need.

This study has some limitations. First, the limited number of interviews (GPs) may not capture the full diversity of GP perspectives, which restricts the range of user needs identified and may reduce the generalisability of the design insights. However, it is important to clarify that the aim was not to achieve data saturation, as would typically be expected in grounded theory or other purely qualitative methodologies. Instead, as the dashboard was developed using the ADR approach, the emphasis was on iterative problem-solving and ongoing refinement. In this context, data saturation was not a primary objective, as ADR prioritises practical relevance and continuous GPs’ engagement (the same GPs participated in both rounds of interviews) over comprehensive data collection.

Second, although the iterative design process was pragmatic and consistent with ADR principles, it may have introduced researcher influence during the interpretation of feedback, potentially affecting objectivity. Finally, the lack of real-world implementation and evaluation of the dashboard means that its usability and effectiveness in practice remain untested.

However, the CARA dashboards and infrastructure were launched in early 2024.^25^ Since then, several actions have been undertaken to generalise the findings. These include exploring the technical feasibility of the CARA infrastructure and dashboards in real-world settings and conducting a user experience study with GPs and researchers using synthetic data (DEMO dashboard).^38^

The next steps will be to monitor dashboard performance and consistency, which is essential in maintaining data integrity to ensure the dashboard functions efficiently and effectively. GPs’ engagement will be measured through the use of different initial dashboard messages.^44^ These actions (future work) will ensure the dashboard is useful for all GPs (generalisation of the solution), and a thorough evaluation will support the derivation of design principles applicable to dashboards in general practice as part of ‘*formalisation of learning*’.

## Conclusions

The development of a *useful* dashboard is based on two key elements: users’ requirements and their continued involvement in the development of the content and overall design decisions. This bottom-up approach ensures a practical dashboard for GPs to view their current patient management and identify quality improvement opportunities.

## Supplementary material

10.1136/bmjopen-2024-086677 online supplemental file 1

## Data Availability

Data are available upon reasonable request.
