# Supplementary material for: Action design research to develop an interactive dashboard to visualise and compare patient data from Irish general practice (CARA)
Source: BMJ Open. 2025 Sep 4;15(9):e086677. doi: 10.1136/bmjopen-2024-086677 (PMC12414190; doi:10.1136/bmjopen-2024-086677)
Supplement: online supplemental file 1 [file bmjopen-15-9-s001.docx]

Supplementary material

# Supplementary material 1. Dashboard Questionnaire (workshop)

Today, we presented the CARA dashboard with information on your practice and antibiotic prescribing (combined with an audit). We would love to hear your opinion about this prototype. This will help us with the improvement and implementation of the CARA dashboard in Irish general practice. The questionnaire take about 5 minutes and your responses are completely anonymous.

On a scale of 1-5, how easy is it to navigate the user dashboard?

**Very Easy Very Difficult**

1 2 3 4 5

|  |
| --- |

How often do you think you would use this dashboard?

a. Daily b. Weekly c. 1-2 times a month d. Never. e. For audit purpose only

Other:______________________________________________________________

Was there anything you missed (specific graphs or different filters)?

|  |
| --- |

Which graph or section of the dashboard did you find not useful, if any?

|  |
| --- |

What dashboard/audit (topic) would you like to see next?

| Chronic Disease | ☐ | Vaccination | ☐ |
| --- | --- | --- | --- |
| Mental Health | ☐ | Opioid Prescribing | ☐ |
| Other:______________________________________________________ | | | |

Thank you for your time for completing this questionnaire and attending this workshop. Your opinions matter to us a lot and will help us improve the CARA dashboard prototype. If you have any other comments or suggestions, please let us know by emailing us at: info@CARAnetwork.ie.

# Supplementary table 1. Results of [Interviews 1]: codes and themes

| **Examples of Original Statements** | **Code** | **Theme** |
| --- | --- | --- |
| as an individual practitioner, I think this was a good dashboard. If it was maybe at the national level, it might be confusing to try and figure out what's happening | Levels of users | User Context |
| it's important to adjust that by some sort of population distribution or, you know, the type of population people see | Practice Context |  |
| Too much prescribing of antibiotics is bad that's what we all sort of agree on, but when you divvy into it or delve into it or think of our world as a connected global world it's probably not as simple as we sort of think. | Making complexity understandable | Sense-making |
| an audit tool based on each system [PMS] | Cross-applicability | Audits |
| the information system […] it's not great for audits or monitoring clinical practice. | Supporting Audits |  |
| how many antibiotics am I prescribing and how does that compare to my peers | Comparing to others | Relevancy |
| split according to medical card or non-medical card | Information Need |  |
| what sort of patients are you seeing, what age groups, what consultations are you providing | Practice Overview |  |
| if I see that I'm prescribing a lot, I would like to see what's going on around, if there is anything circulating in the community | Providing (local) context |  |
| you really do need something that is up to date | Up-to-date information |  |
| I can see this being useful | Usefulness |  |
| if you see the value of using the ICPC codes, I think people will start learning how to do it and to just include that in their daily practice | Encouraging action | Action |
| when I open it up, I get the key message on the first screen | Focus |  |
| how much prescribing was maybe in the guidelines as opposed to out of the guidelines | Following Guidelines |  |
| I tend to pick something that I'll have ongoing anyway and that I’ll find useful | Being interested | Engagement |
| it's about getting data out of around 1500 silos and sort of combining that data and seeing how it can benefit those silos but also how can it benefit the national understanding, public health and research | Combining data |  |
| it's about how the information comes or it could be a prompt in my practice software, you know, it could be on the first of every month a little prompt comes up. | Keeping in touch |  |
| You have to be someone who really wants to learn more about that topic and has time to, so then it's brilliant. | Having time to engage |  |
| you could track it over time | Temporal data |  |
| you much rather see that [visualisations] than numbers | Attractiveness | Ease of Use |
| Even without touching it, it was very easy to see how this might work | Ease of Use |  |
| they're all good and they all had the data that you'd like to see, but some of them just weren't as clear | Clarity |  |
| that information in my mind is on HSE on antibioticprescribing.ie and so I don't think it would change my practice | Avoiding duplication |  |
| it's about not having too much information | Information overload |  |
| I can click back into it and find more detail | Layers of information |  |

# Supplementary table 2. Visual evidence for addressed seven challenges

Dashboard link versions:

1. CARA dashboard prototype v0.1: <https://www.figma.com/proto/6YgVhnHJGF2w7kMPj1Z241/Original-CARA-Prototype?scaling=scale-down-width&page-id=0%3A1&starting-point-node-id=2%3A2&node-id=399-726&hide-ui=1>
2. CARA dashboard prototype v0.2: <https://www.figma.com/proto/0skpQCm3P9DhsvMq6wKBq8/CARA-Dashboard-Prototype-1?page-id=0%3A1&node-id=2-2&p=f&viewport=107%2C210%2C0.03&t=2XCUaQud38KTvCzY-1&scaling=scale-down-width&content-scaling=fixed&starting-point-node-id=2%3A2&hide-ui=1>
3. CARA dashboard prototype v0.3: <https://www.figma.com/proto/sFch8W2K7o8dllamJ46kJy/CARA-Dashboard-Prototype-final?node-id=2-2&scaling=scale-down-width&page-id=0%3A1&starting-point-node-id=2%3A2&hide-ui=1>
4. CARA dashboard prototype v0.4: <https://www.figma.com/proto/X5jC1Z2IlkqVups5YlSK9G/CARA-Dashboard-Prototype-Interactions?page-id=0%3A1&node-id=2-2&p=f&viewport=160%2C245%2C0.02&t=97Dj5Q13r64asOwY-1&scaling=scale-down-width&content-scaling=fixed&starting-point-node-id=2%3A2&hide-ui=1>
5. CARA dashboard v1.0: <https://demo.caranetwork.ie> it was created specifically for the workshop and is no longer available, however, it has been replaced our current DEMO version, which contains synthetic practice data.

| **Link version** | **Context**: Dashboard design needs to adjust dynamically to the user's context to acknowledge that GP practices operate in different contexts, with respect to size, location, age profiles etc. |
| --- | --- |
| **1** | 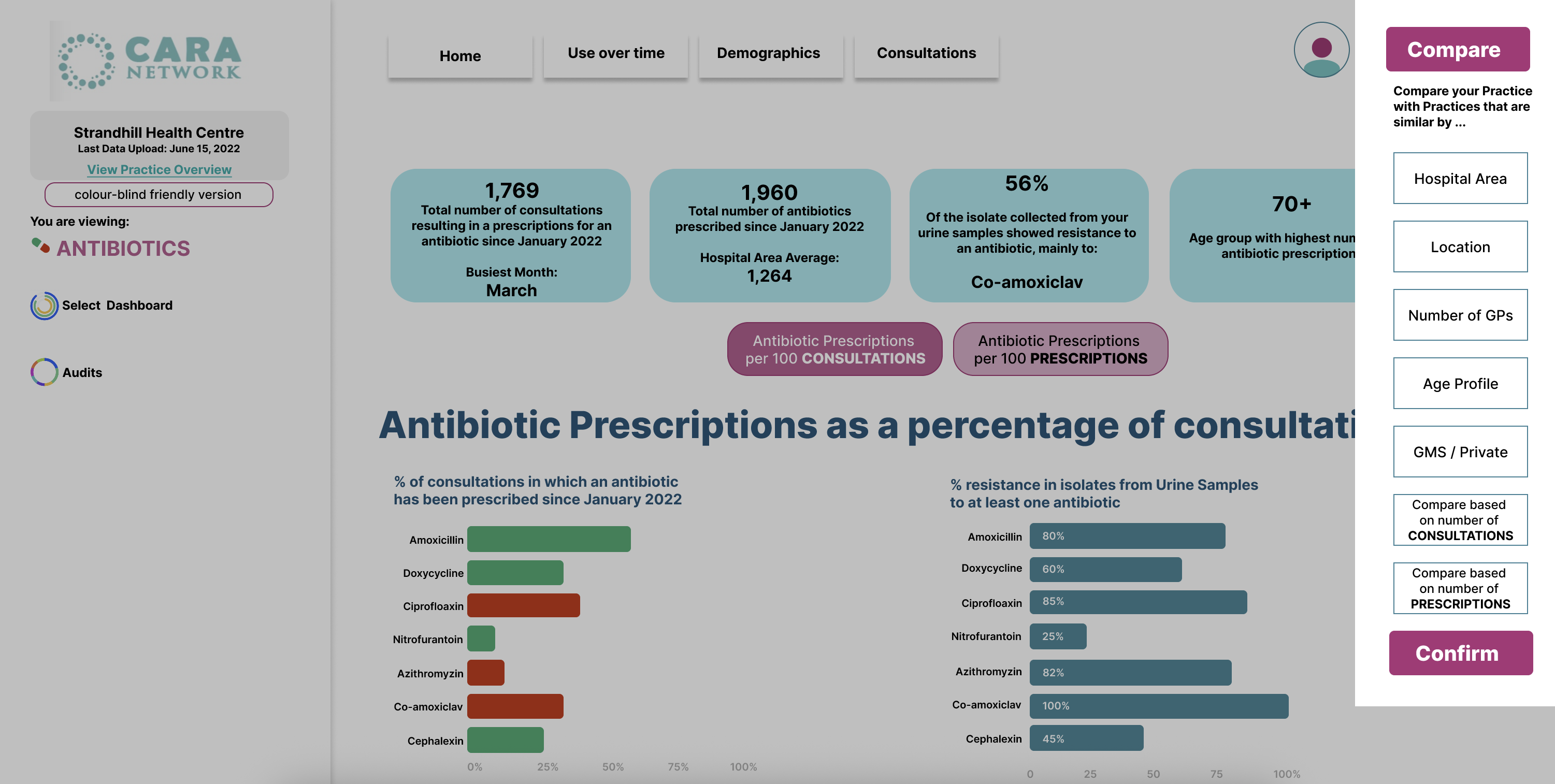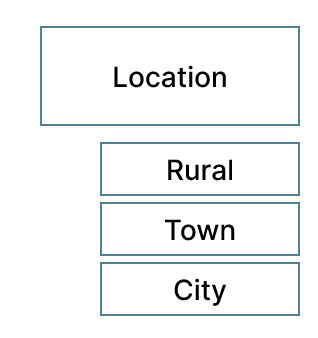 |
| **3** | 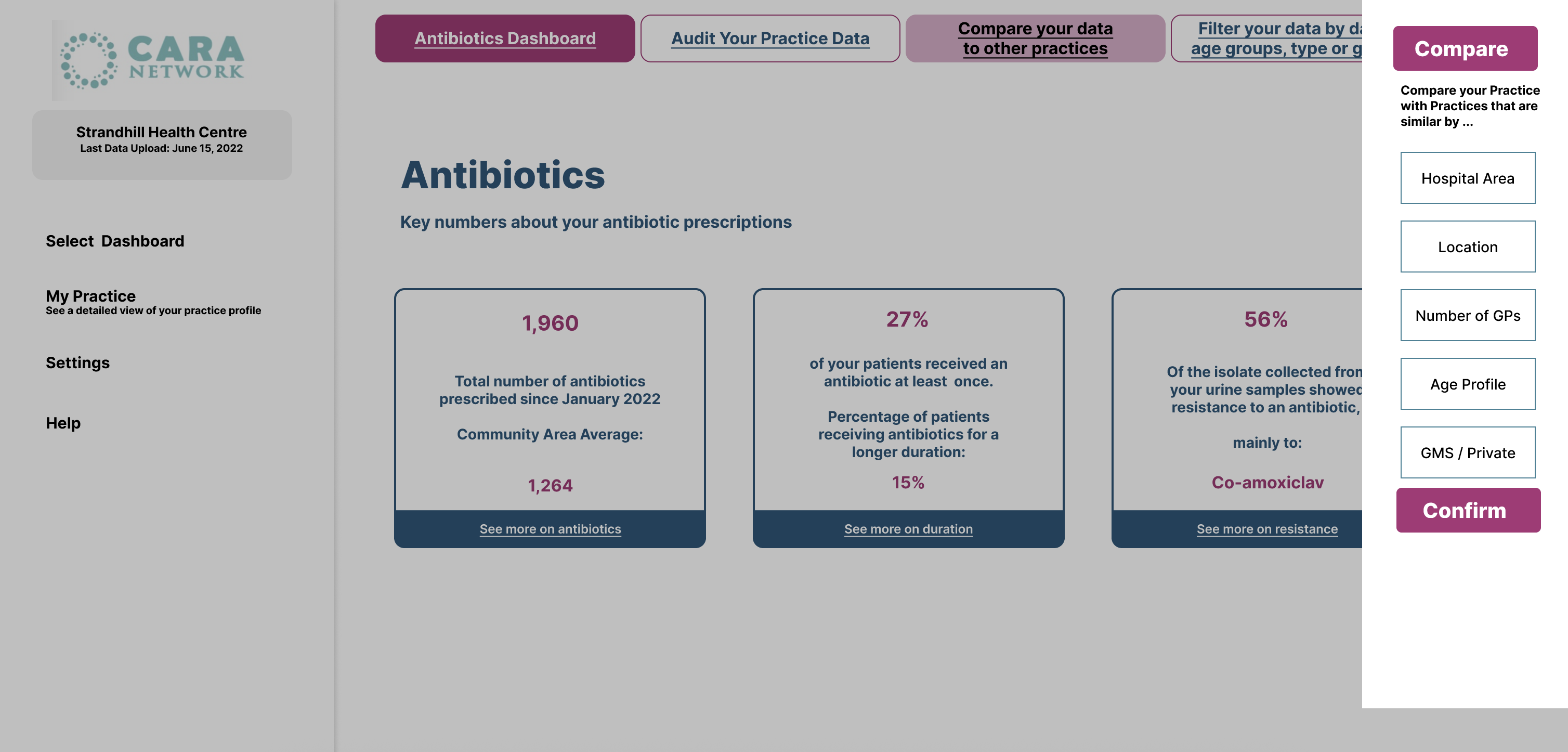 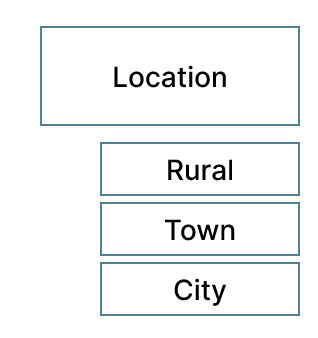 |
| **5** | 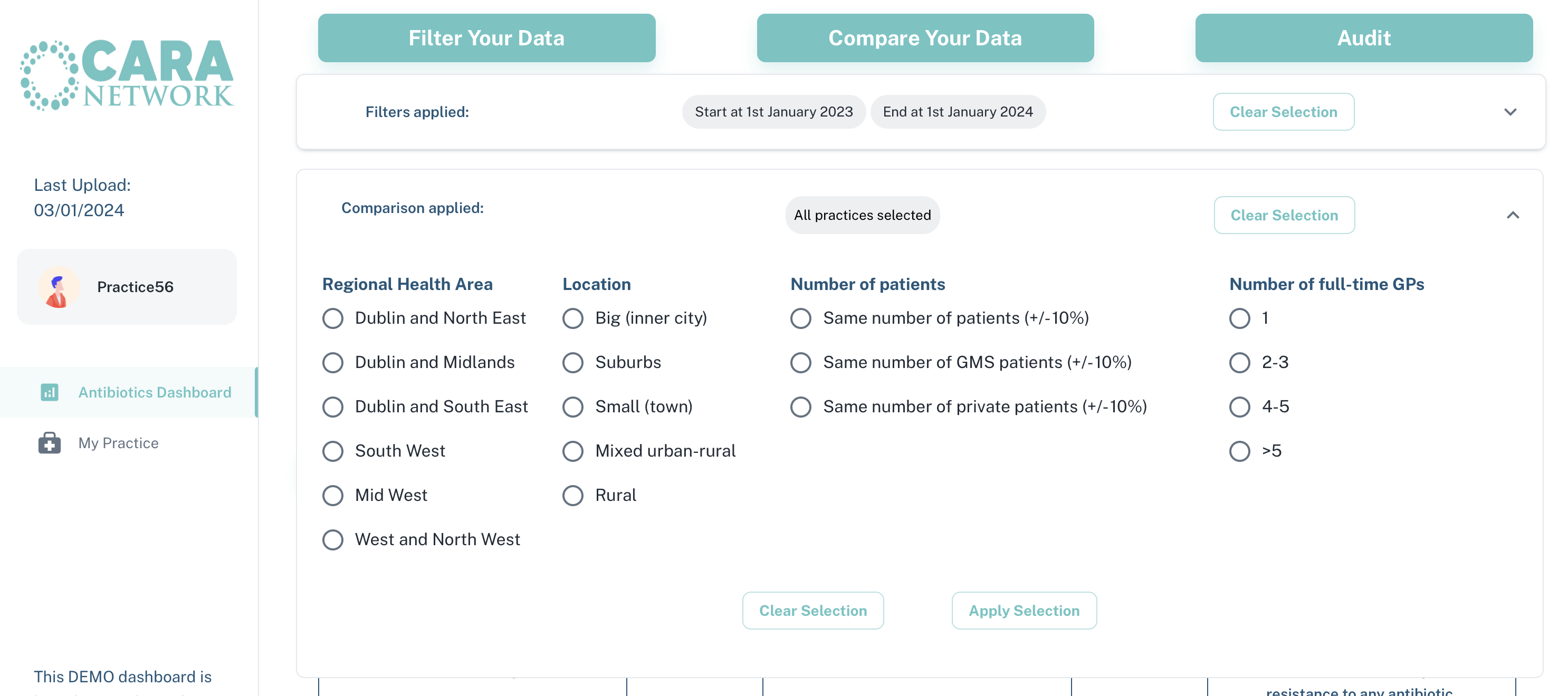 |

| **Link version** | **Context**: Dashboard design needs to adjust dynamically to the user's context to acknowledge that GP practices operate in different contexts, with respect to size, location, age profiles etc. |
| --- | --- |
| **1** | 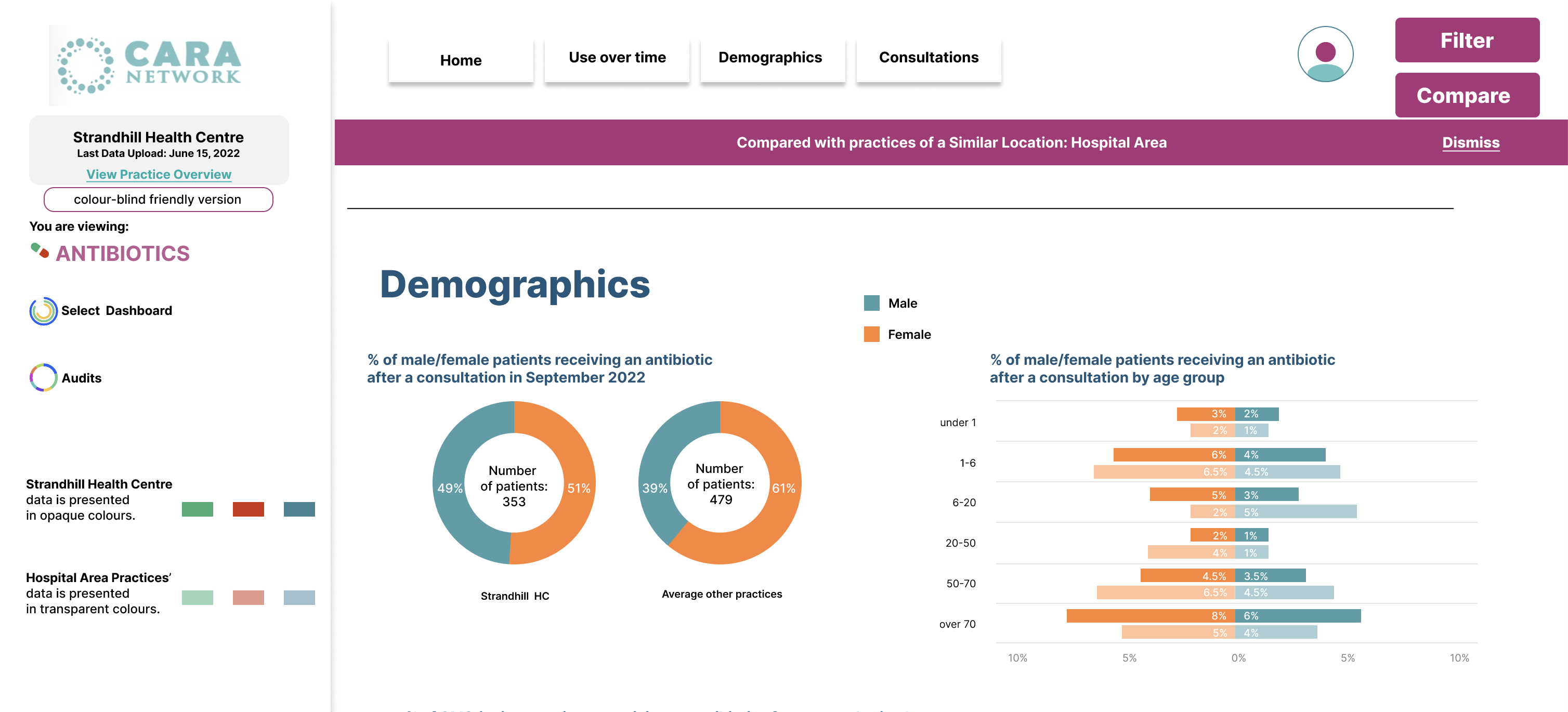 |
| **3** | 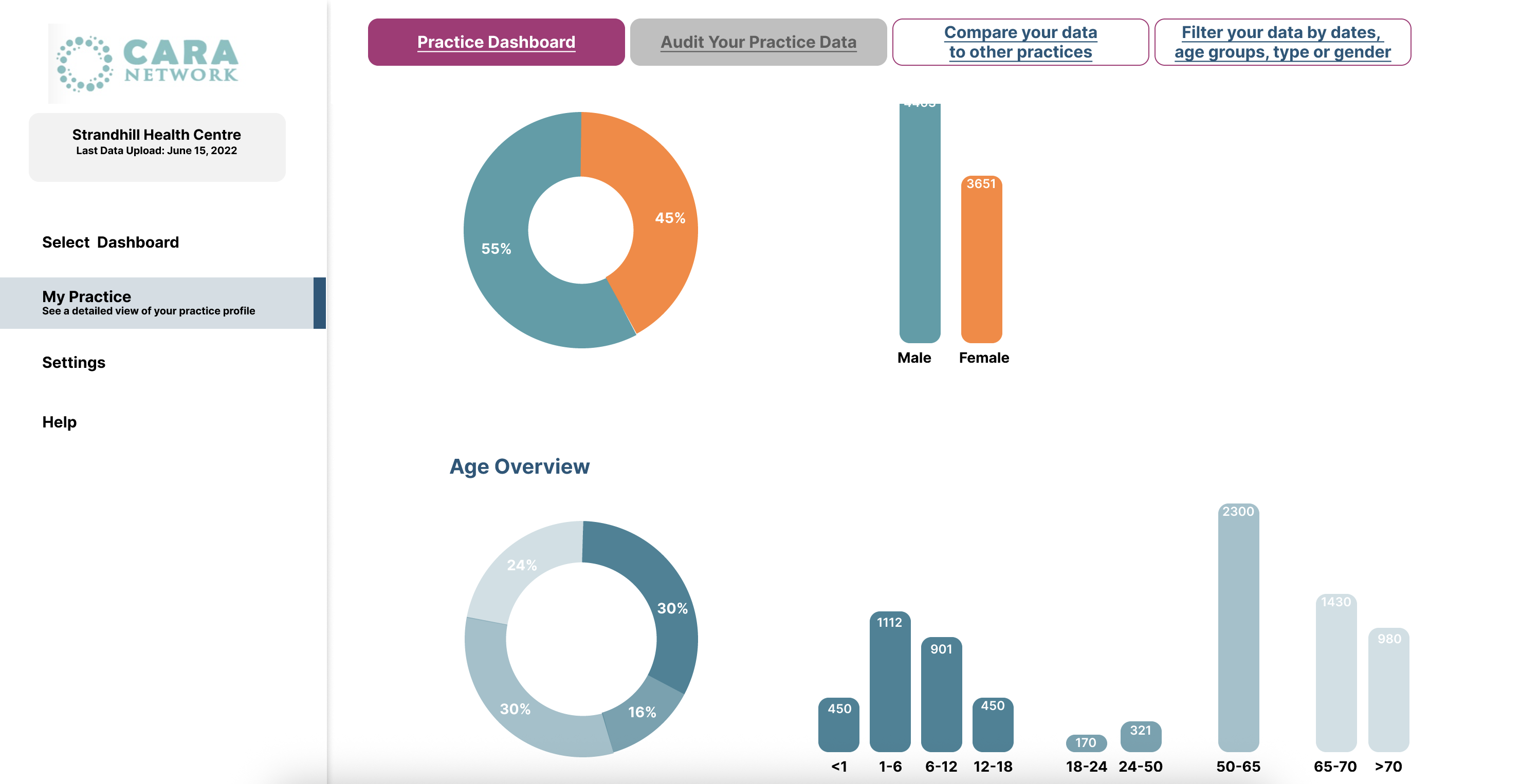 |
| **5** | 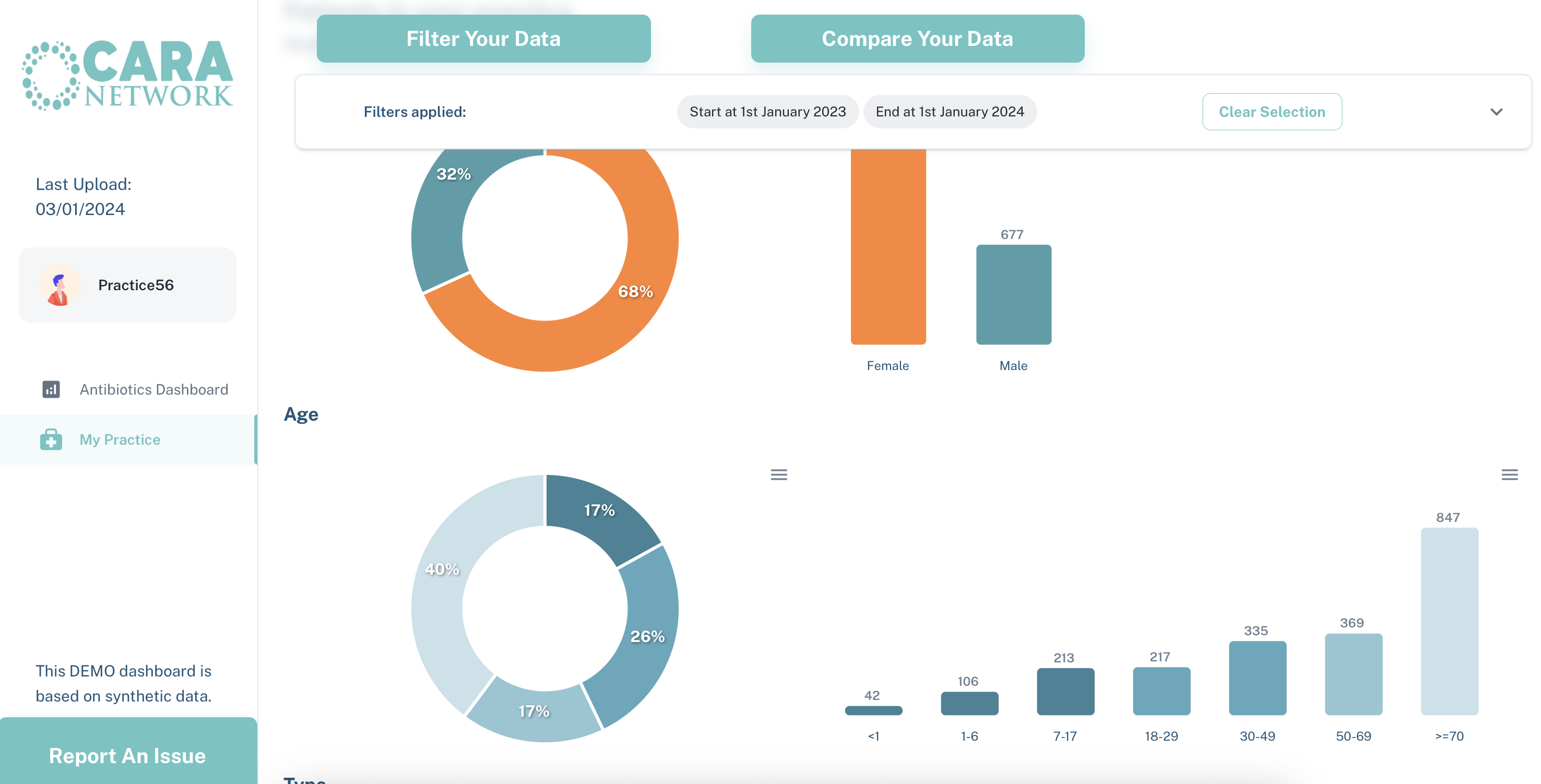 |

| **Link version** | **Sense-making**: Information presented must be understandable. Given the potential volume of information available, information presentation needs to be focused and clear. |
| --- | --- |
| **1** | 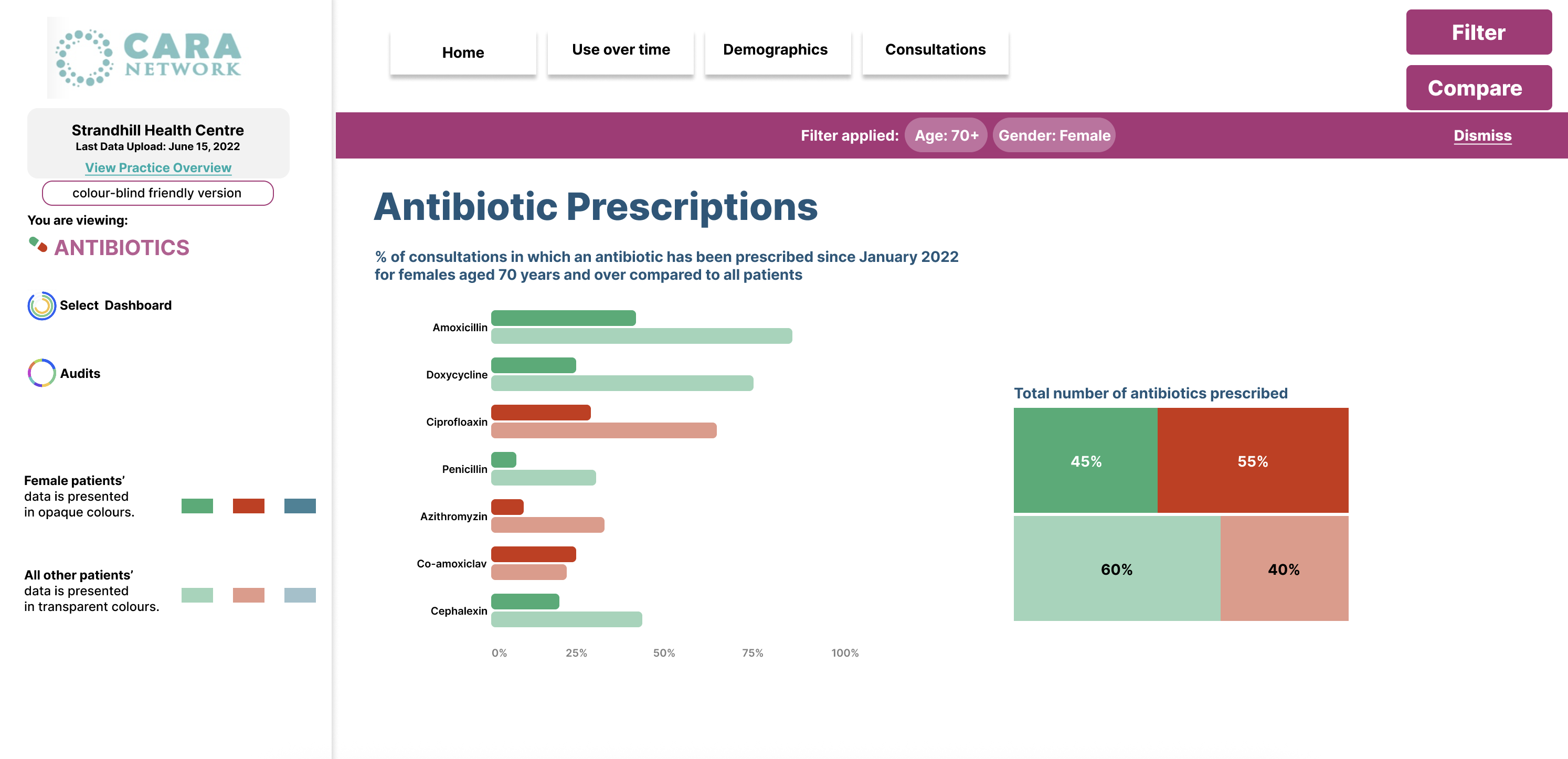 |
| **3** | 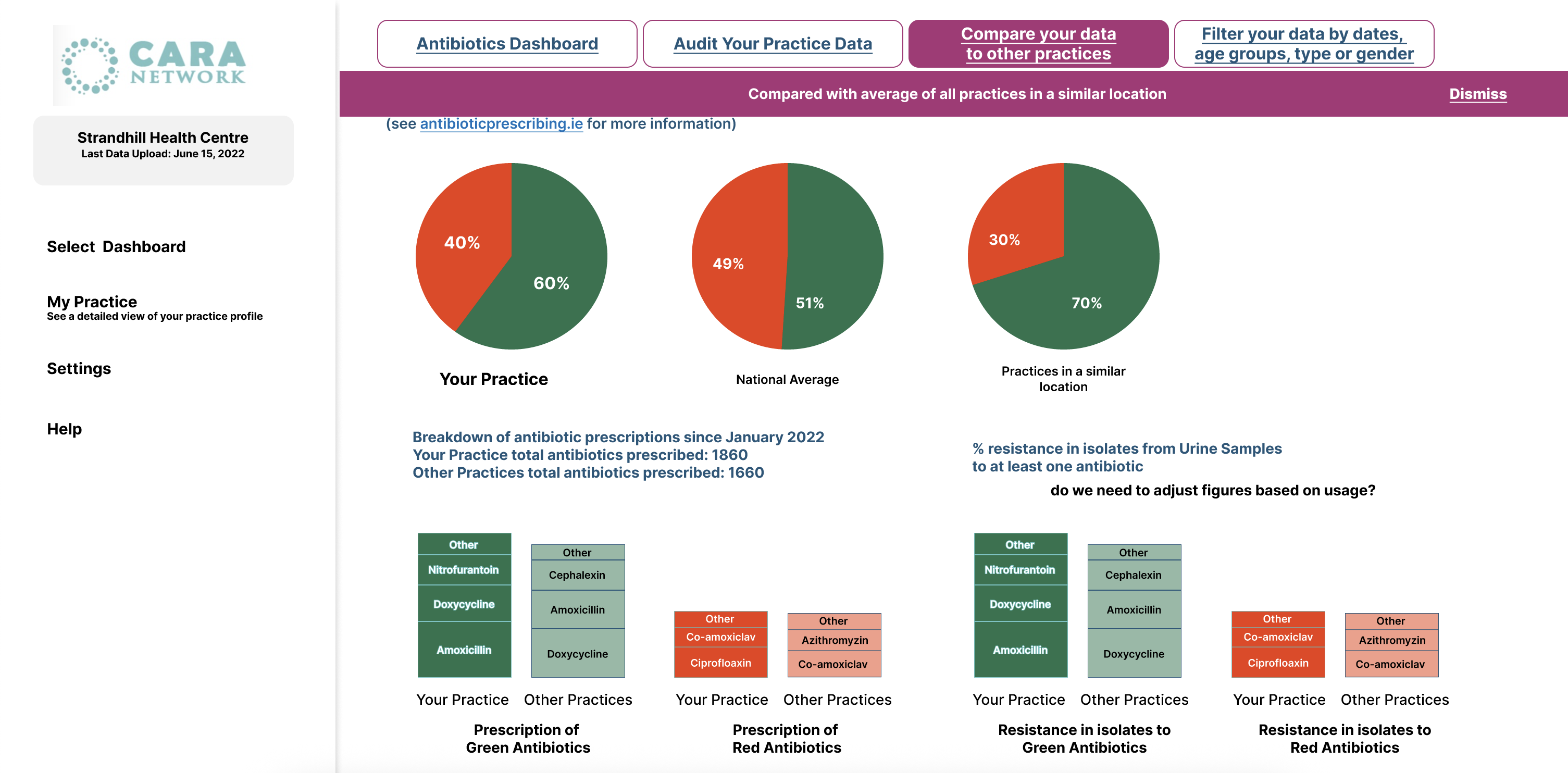 |
| **5** | 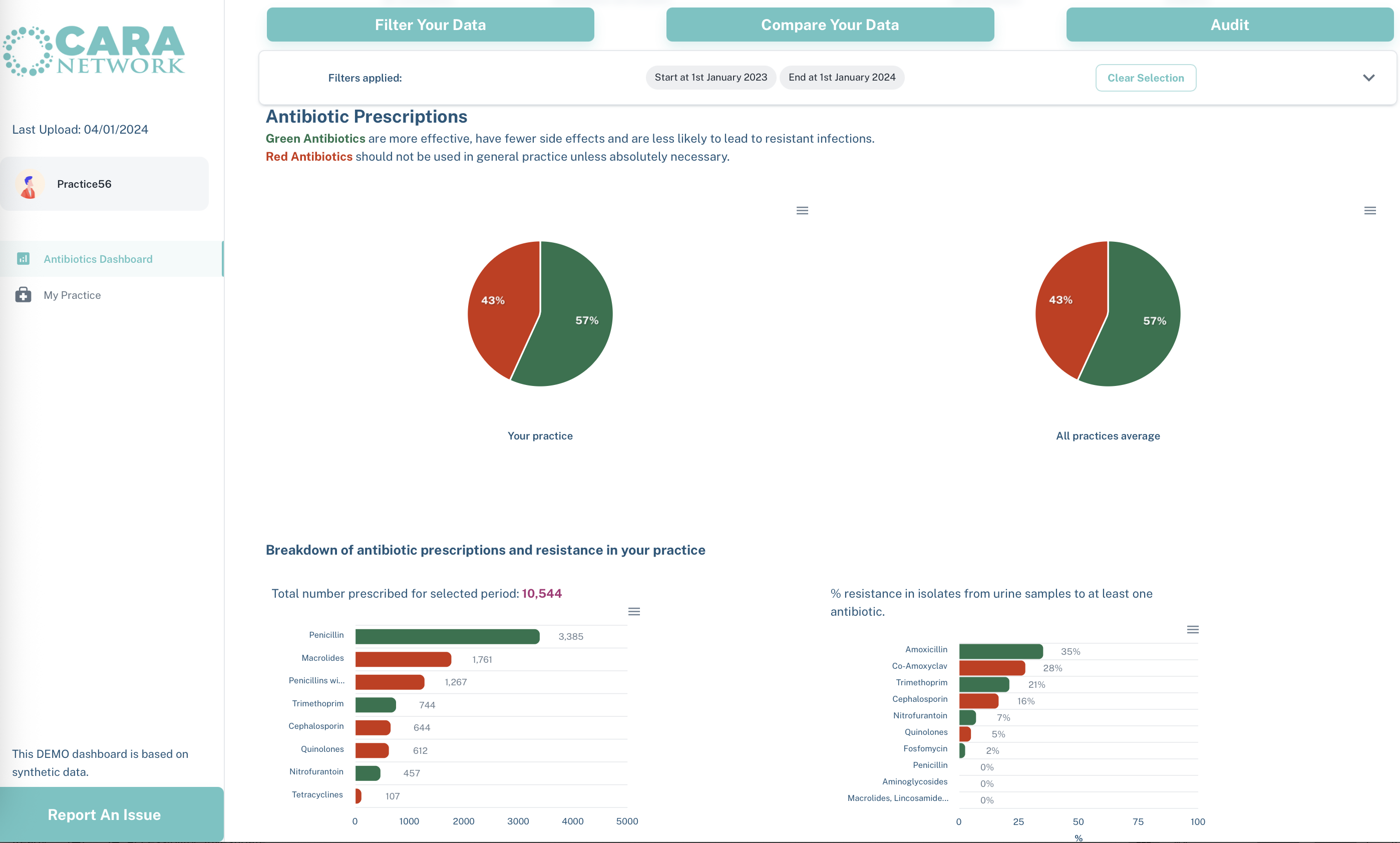 |

| **Link version** | **Sense-making**: Information presented must be understandable. Given the potential volume of information available, information presentation needs to be focused and clear. |
| --- | --- |
| **1** | 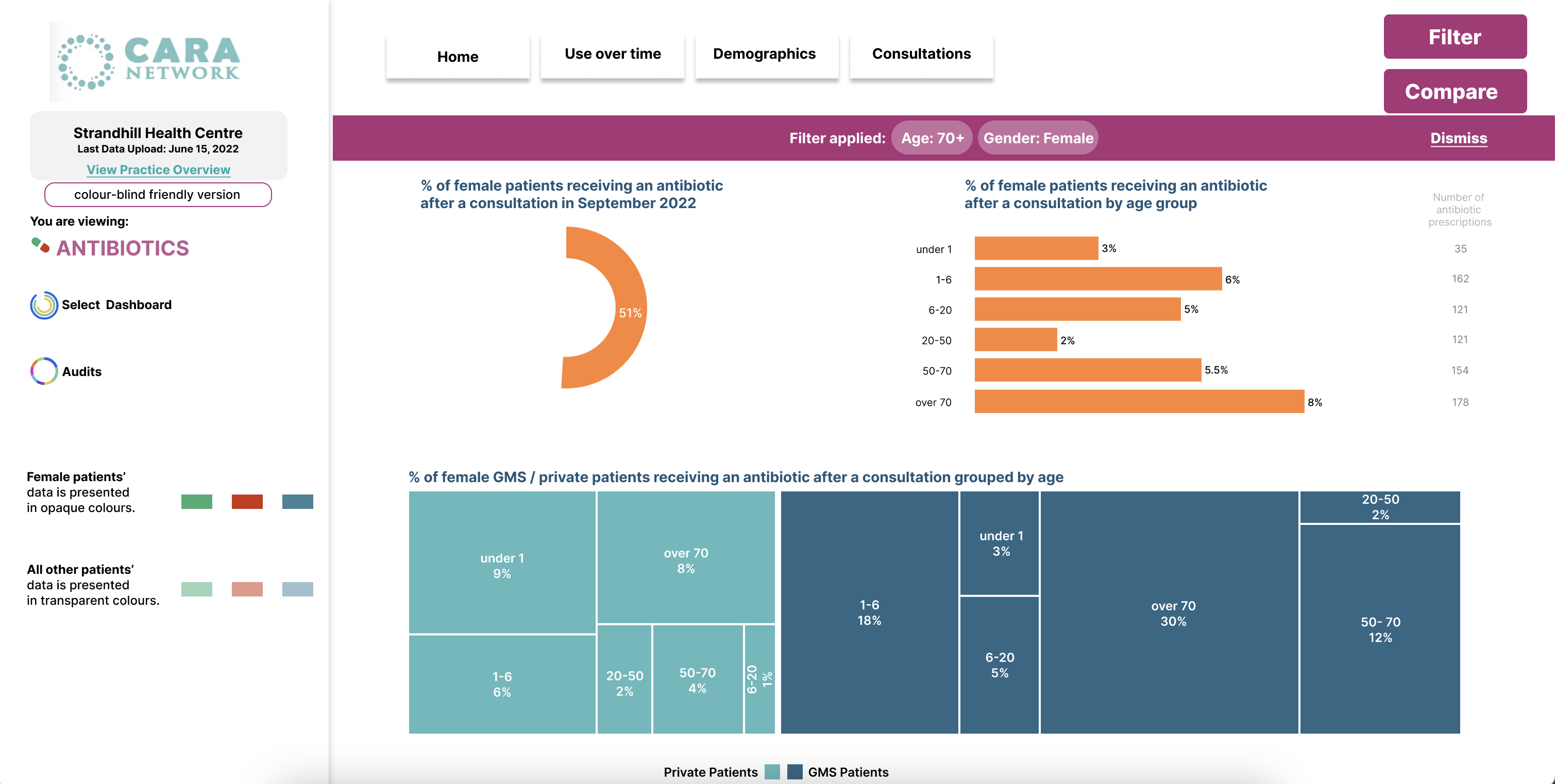 |
| **3** | 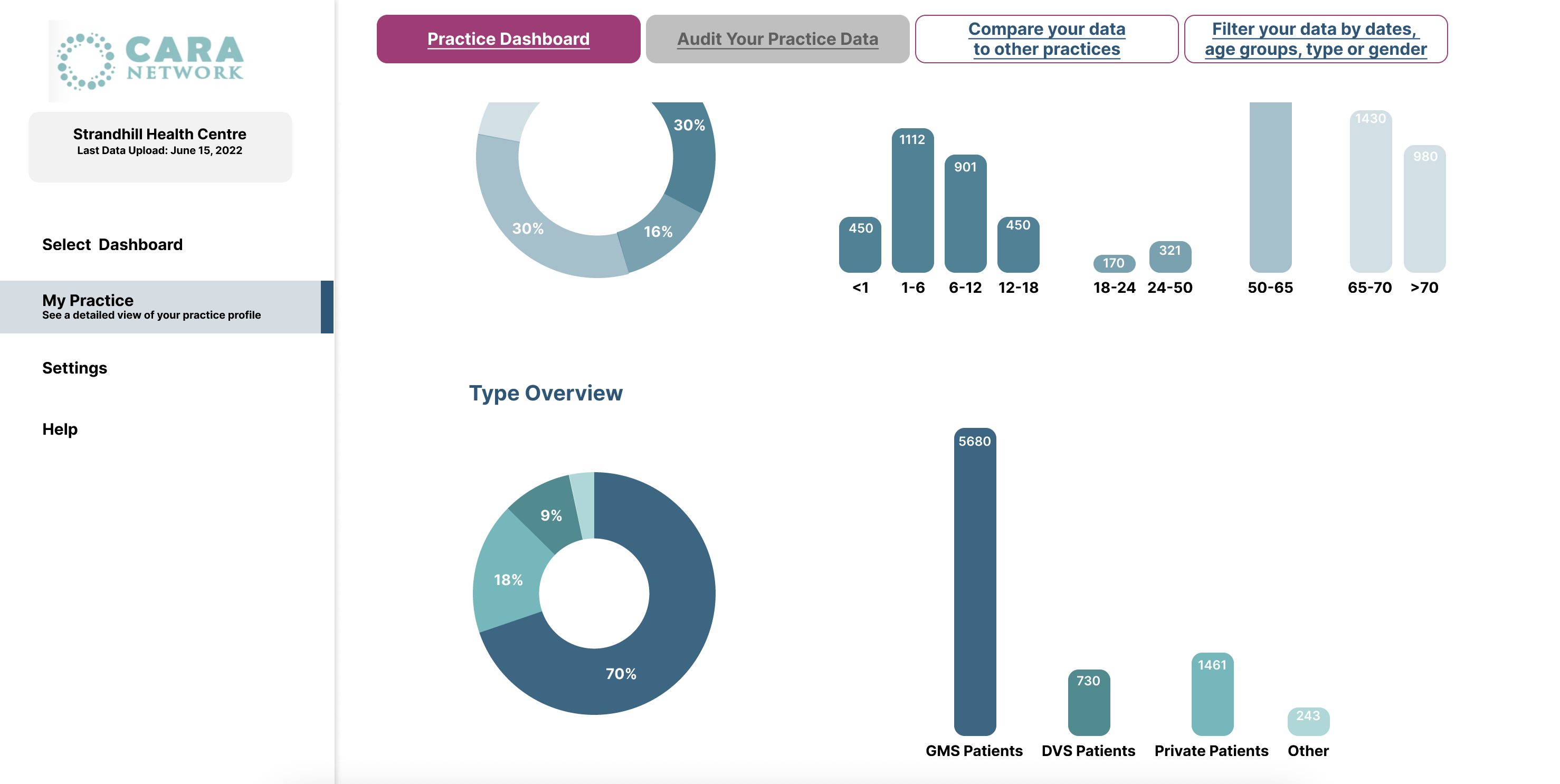 |
| **5** | 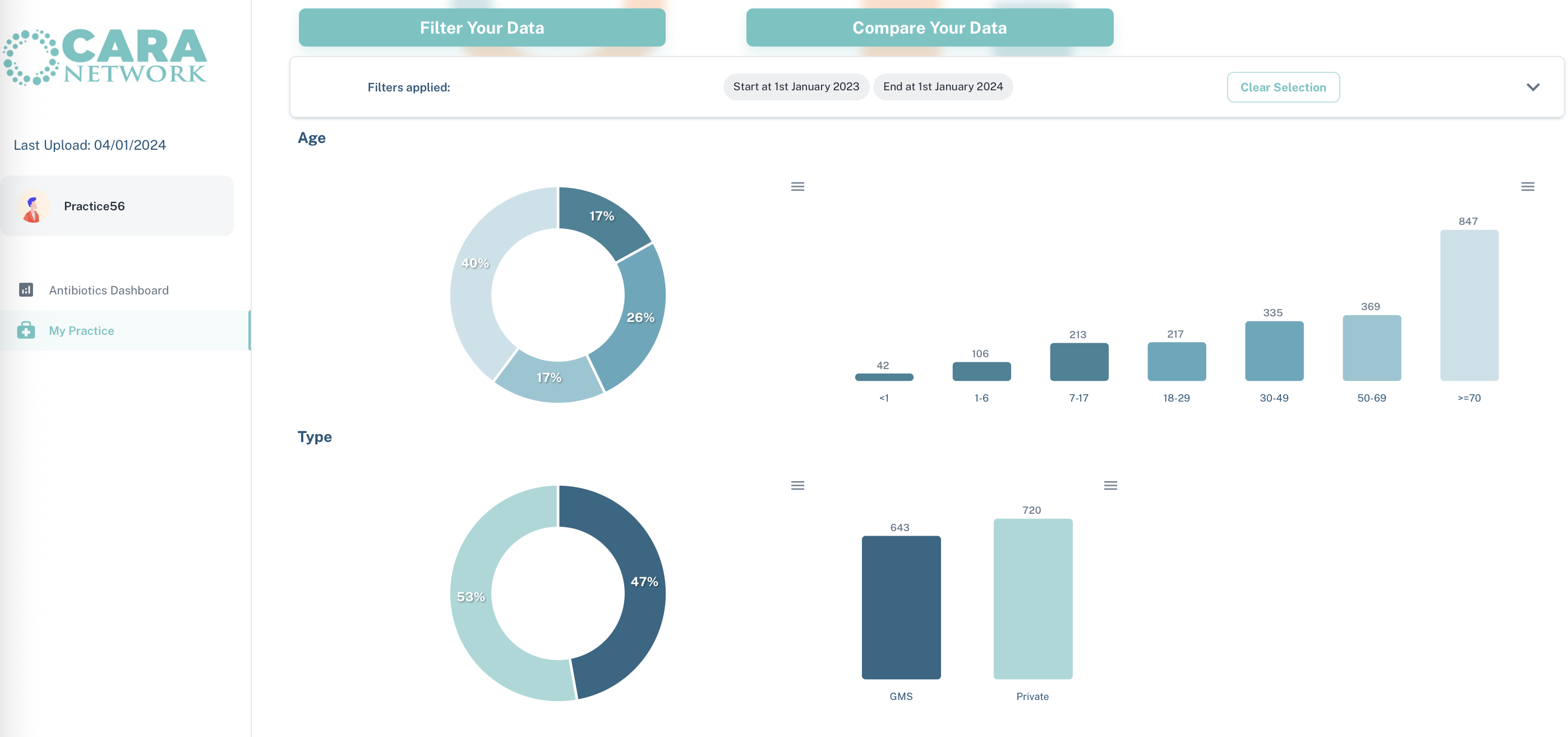 |

| **Link version** | **Audits**: Support to carry out mandatory audits is crucial. Annual audits are a requirement for all GPs and easy generation of audit reports can save GPs time, thereby offering an additional bonus. |
| --- | --- |
| **1** | 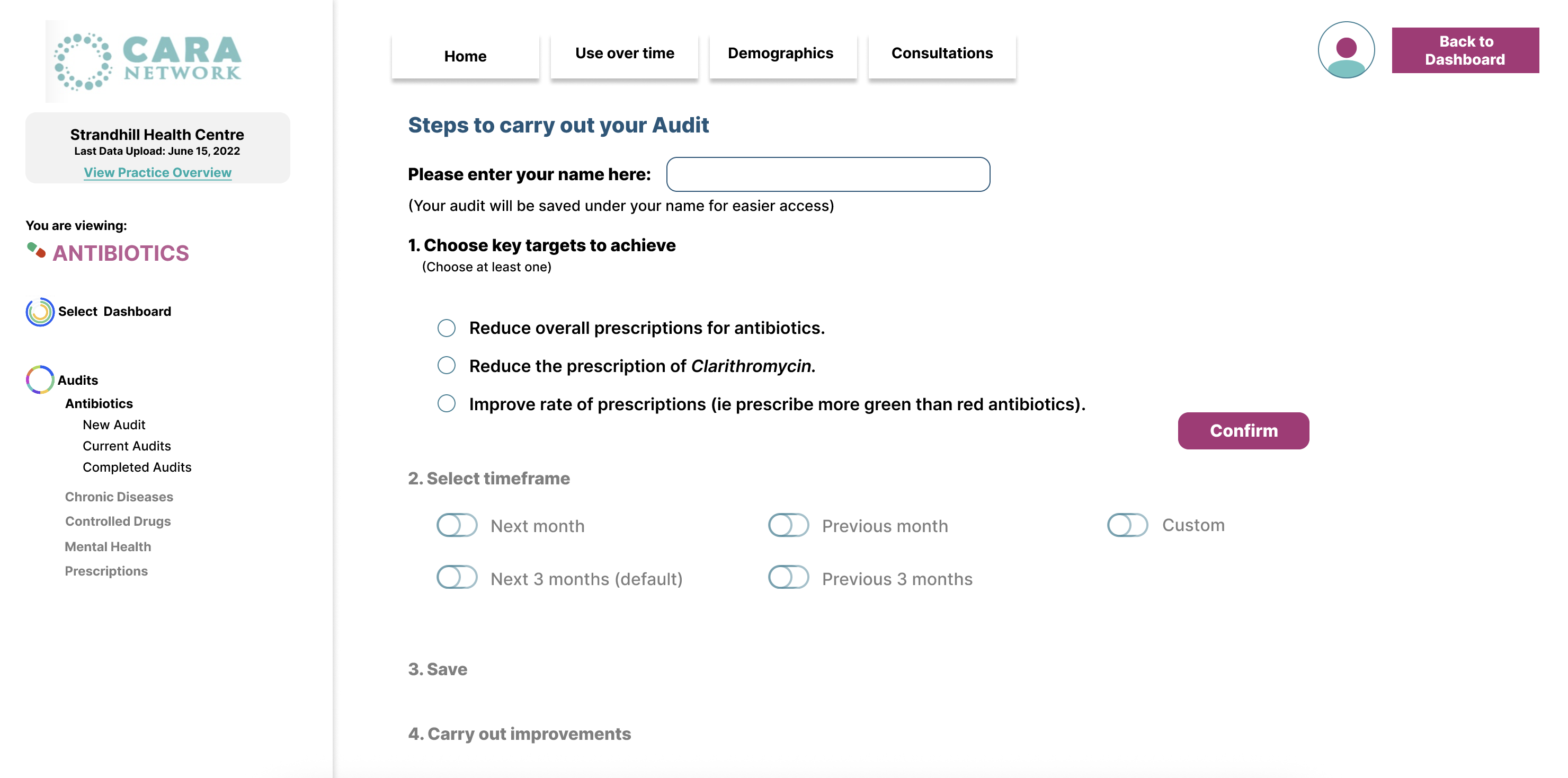 |
| **3** | **Step 2:** 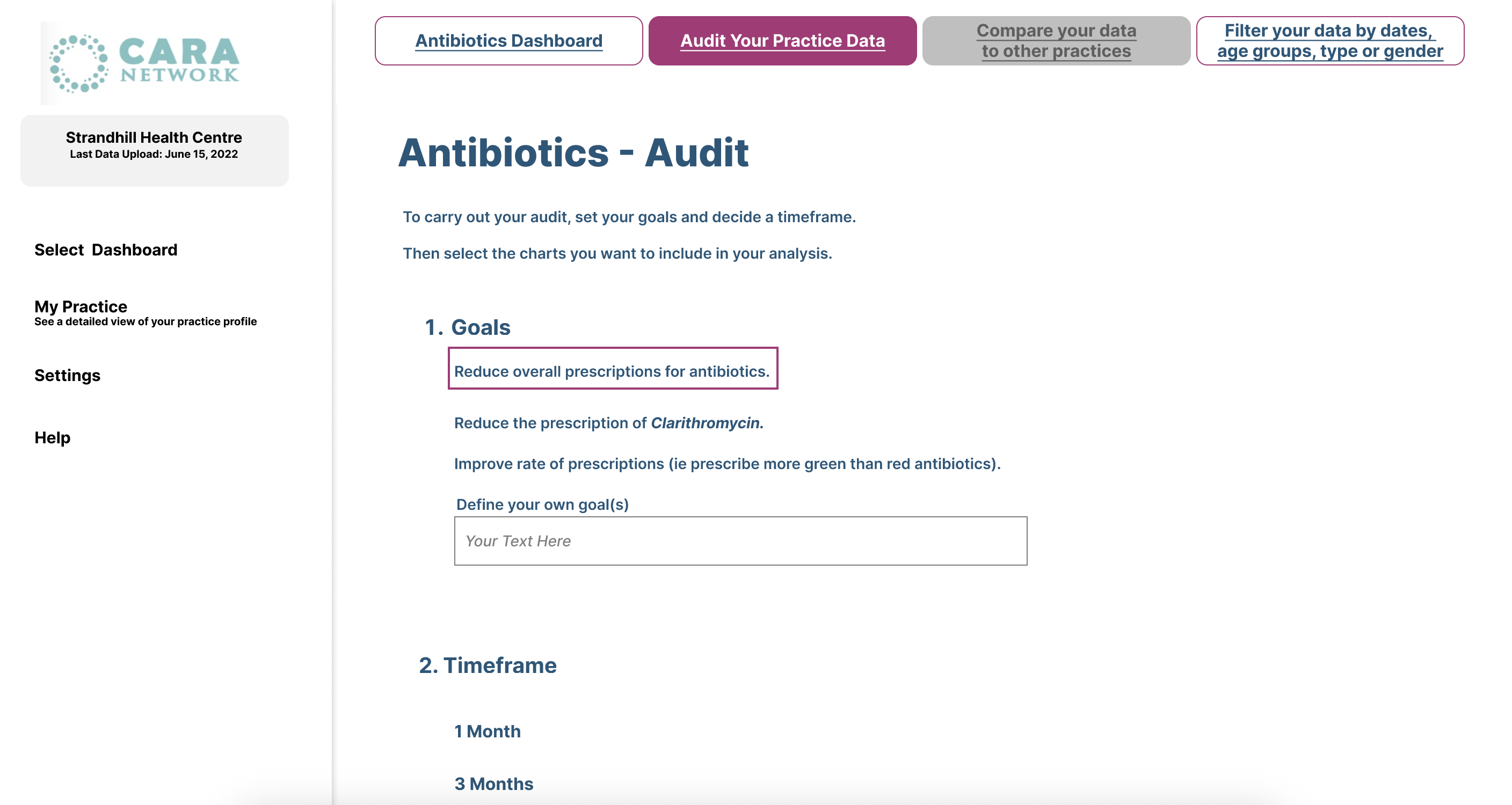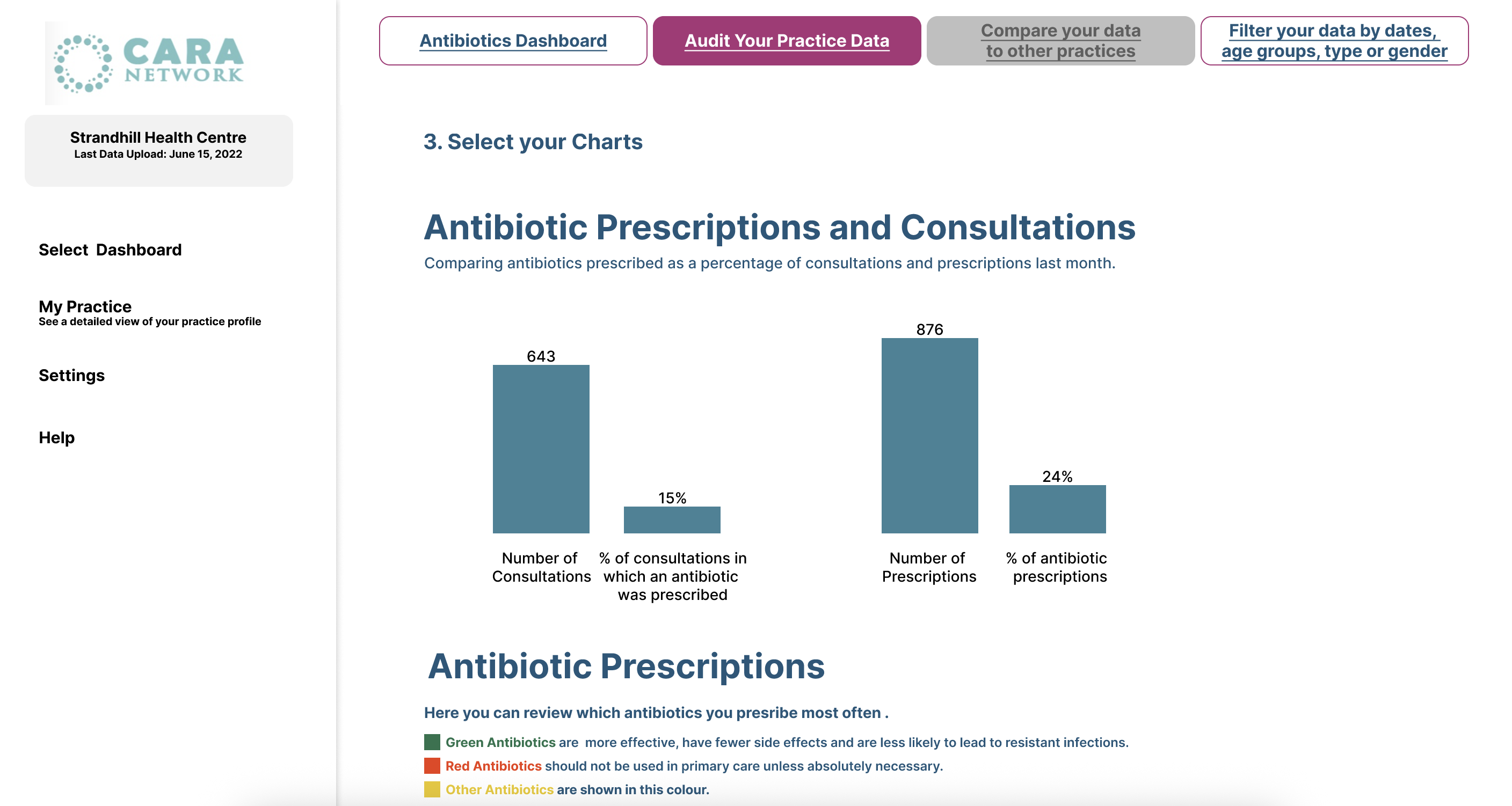 |
| **5** | 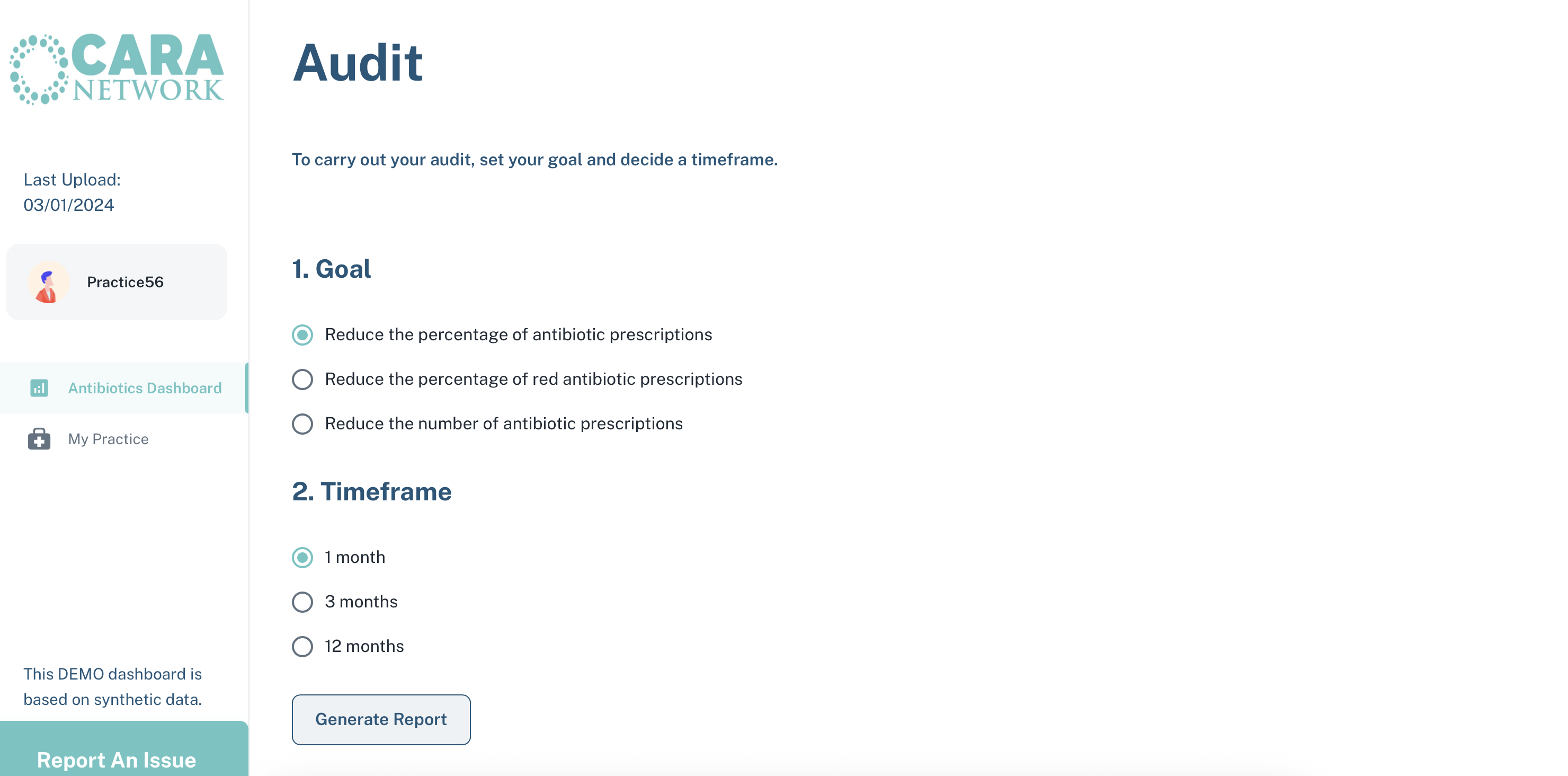Step 2: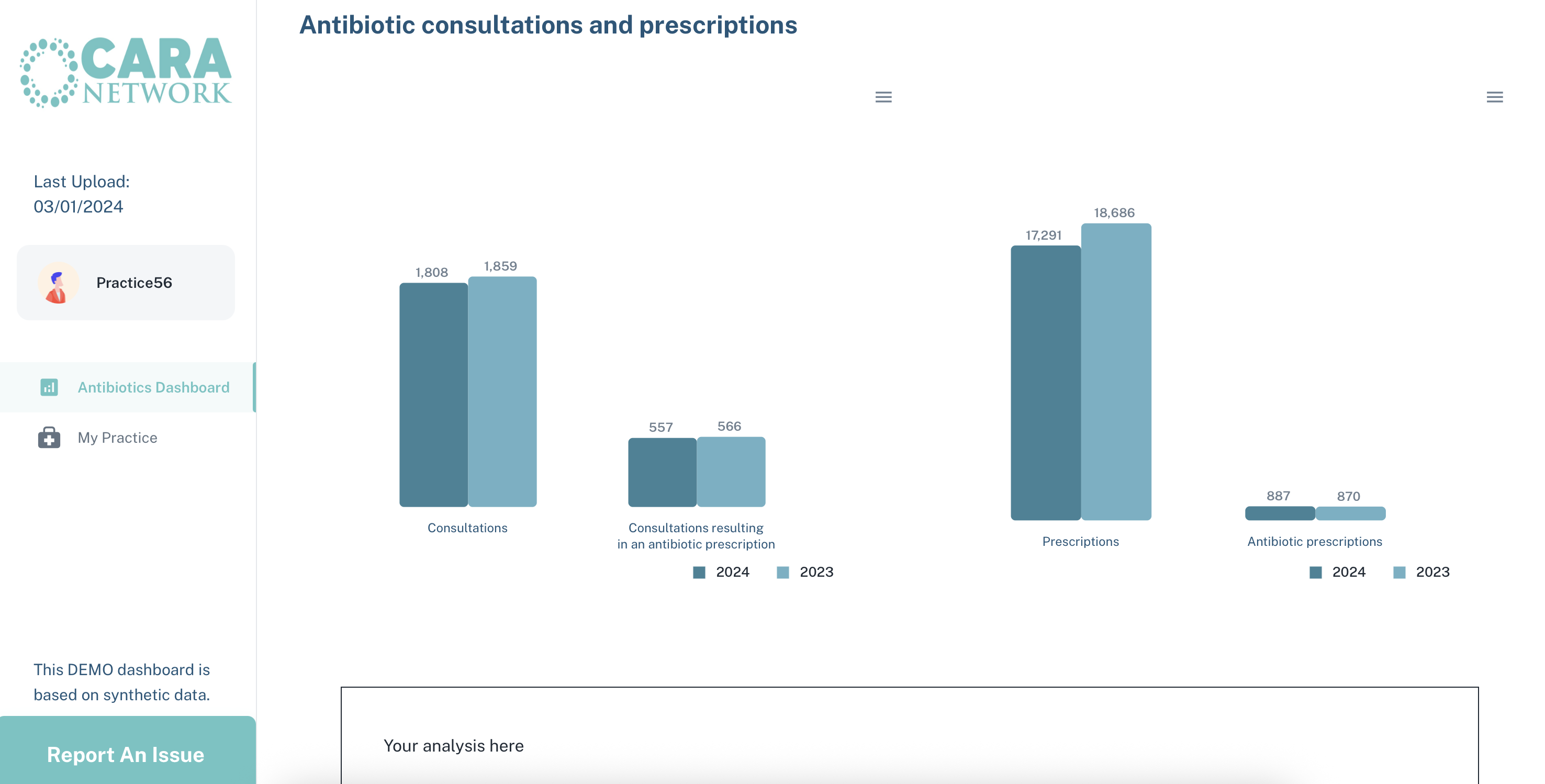 |

| **Link version** | **Relevancy**: The dashboard must be relevant to its users. Data presented on the dashboard must be timely and relevant to the local context. |
| --- | --- |
| **1** | 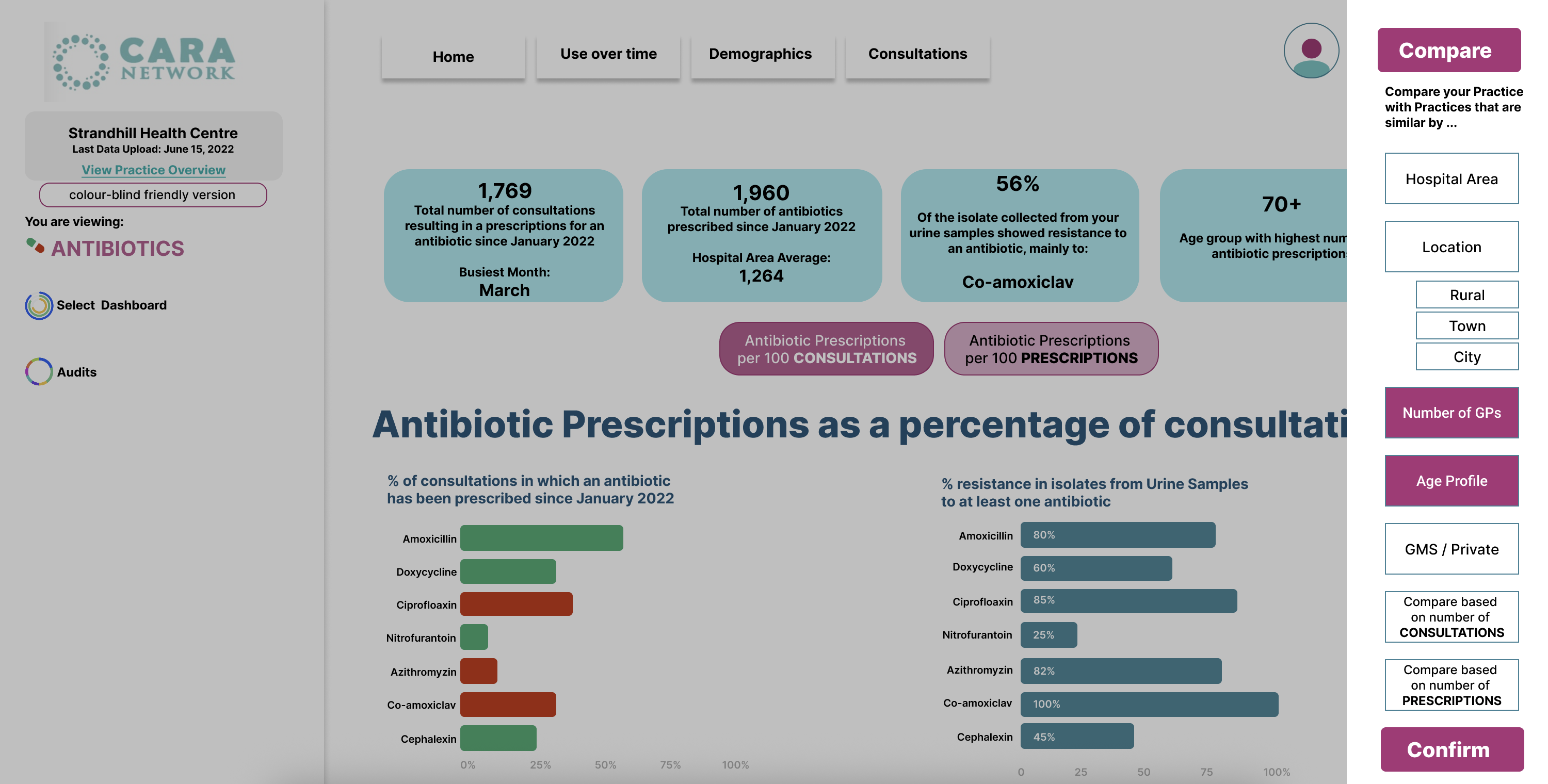 |
| **3** | 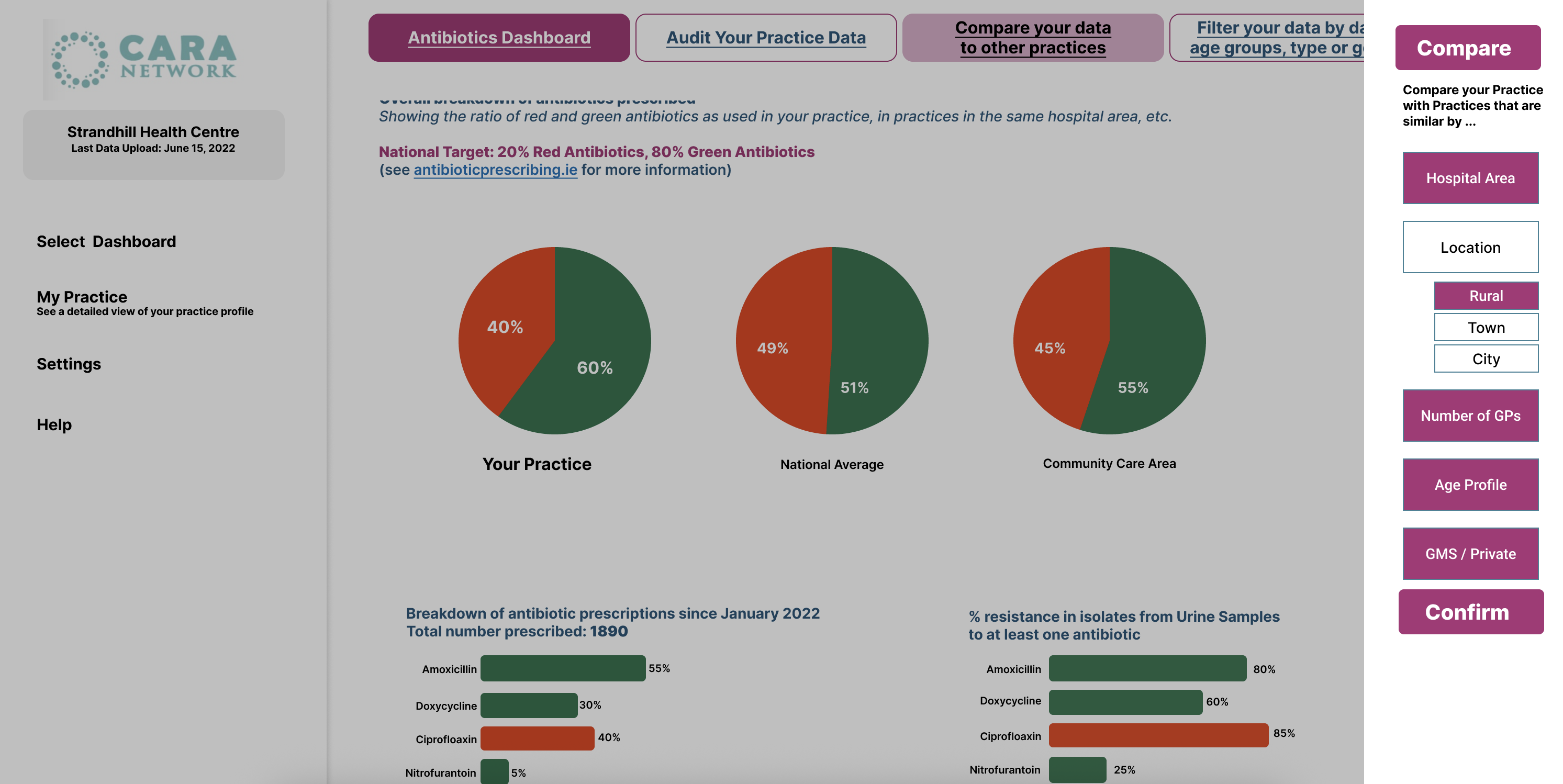 |
| **5** | 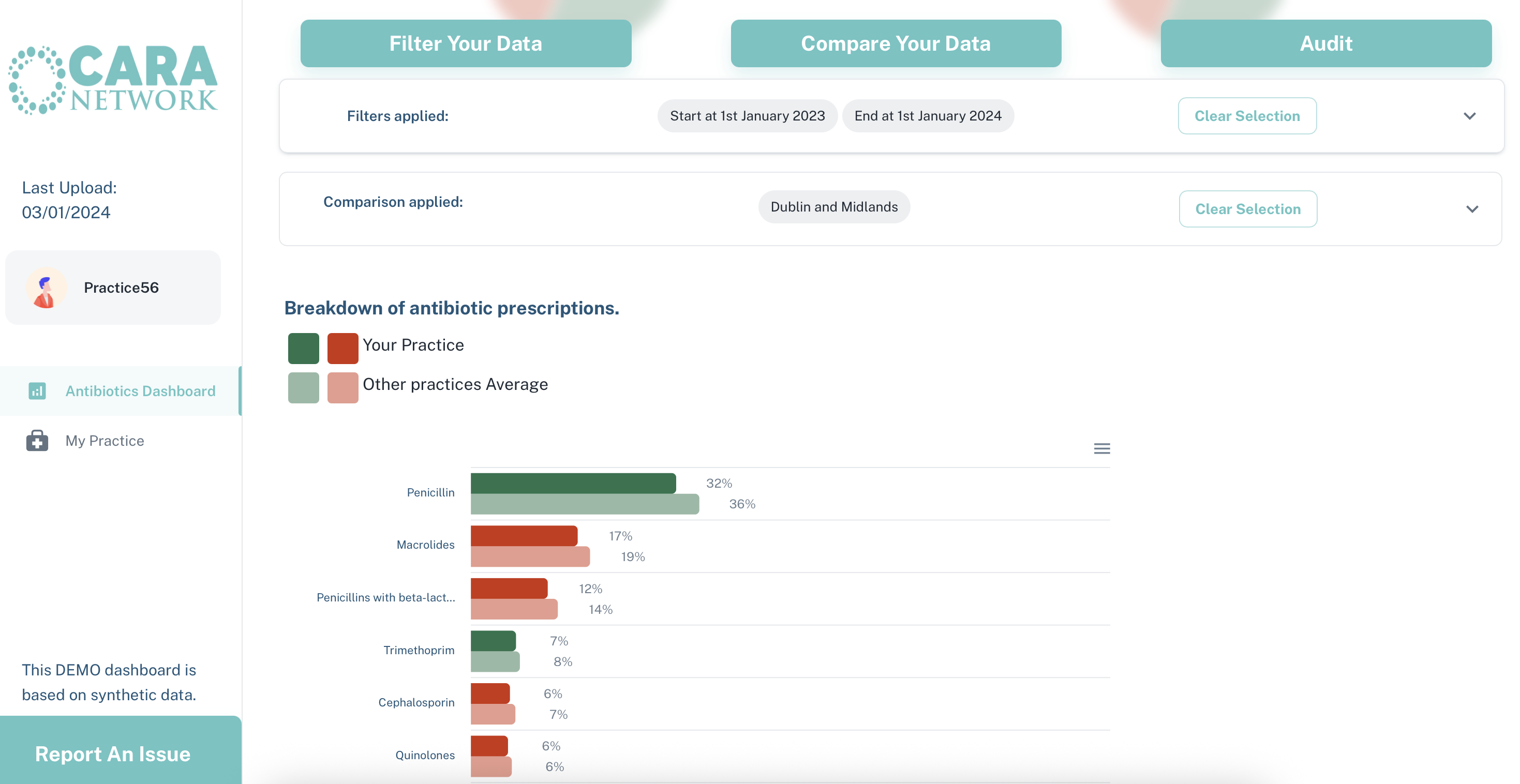 |

| **Link version** | **Relevancy**: The dashboard must be relevant to its users. Data presented on the dashboard must be timely and relevant to the local context. |
| --- | --- |
| **1** | 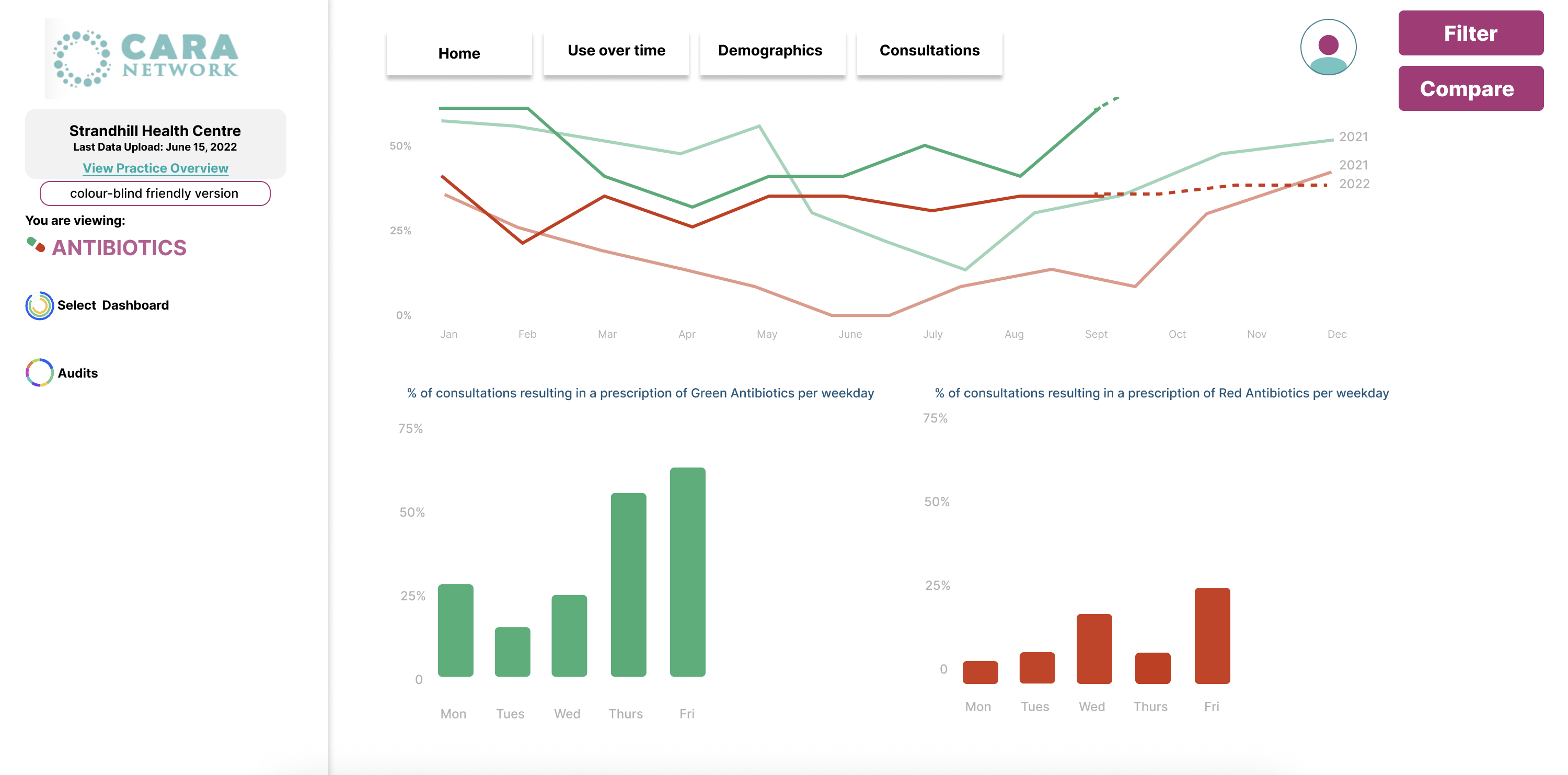 |
| **3** | 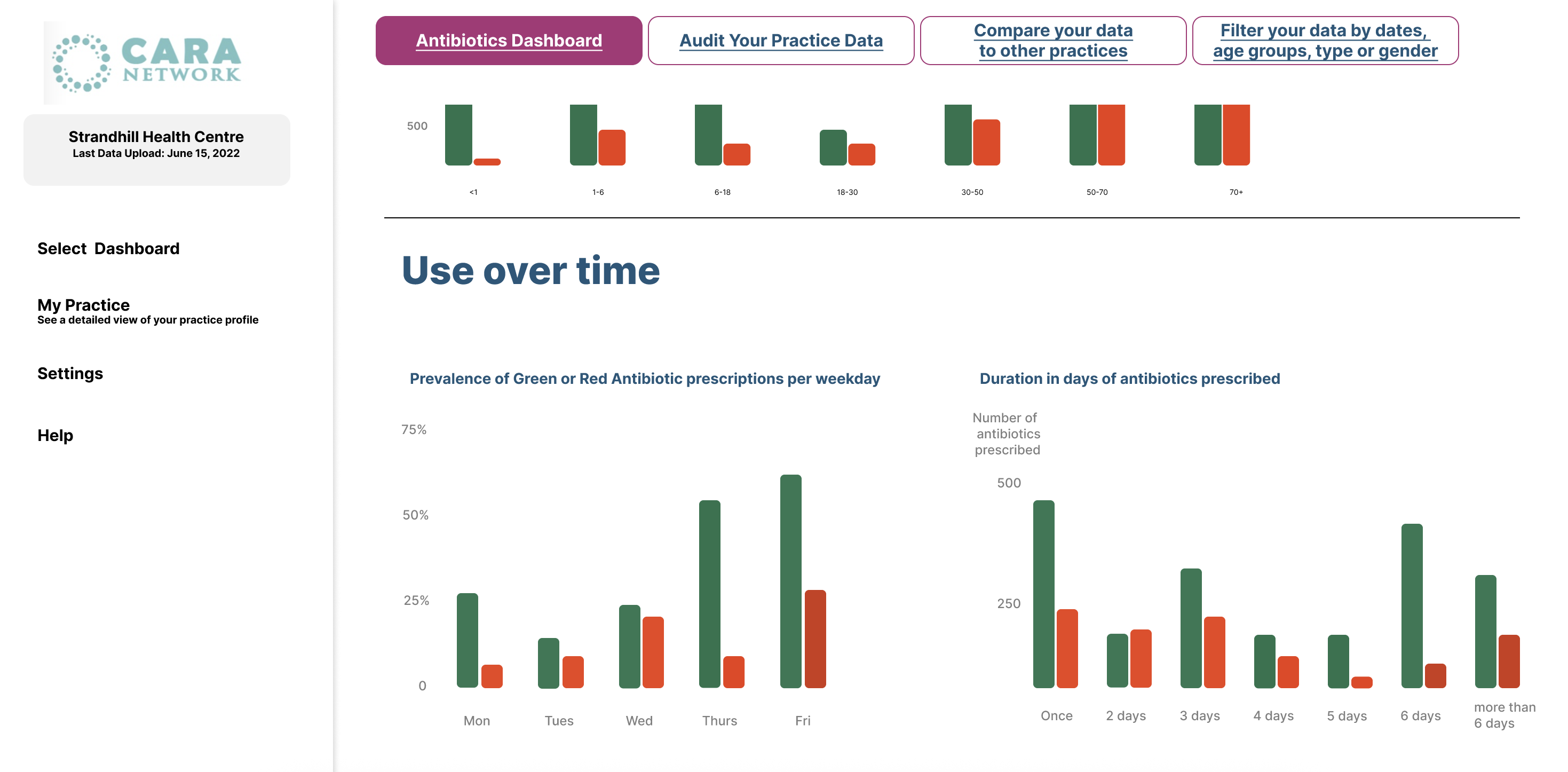 |
| **5** | 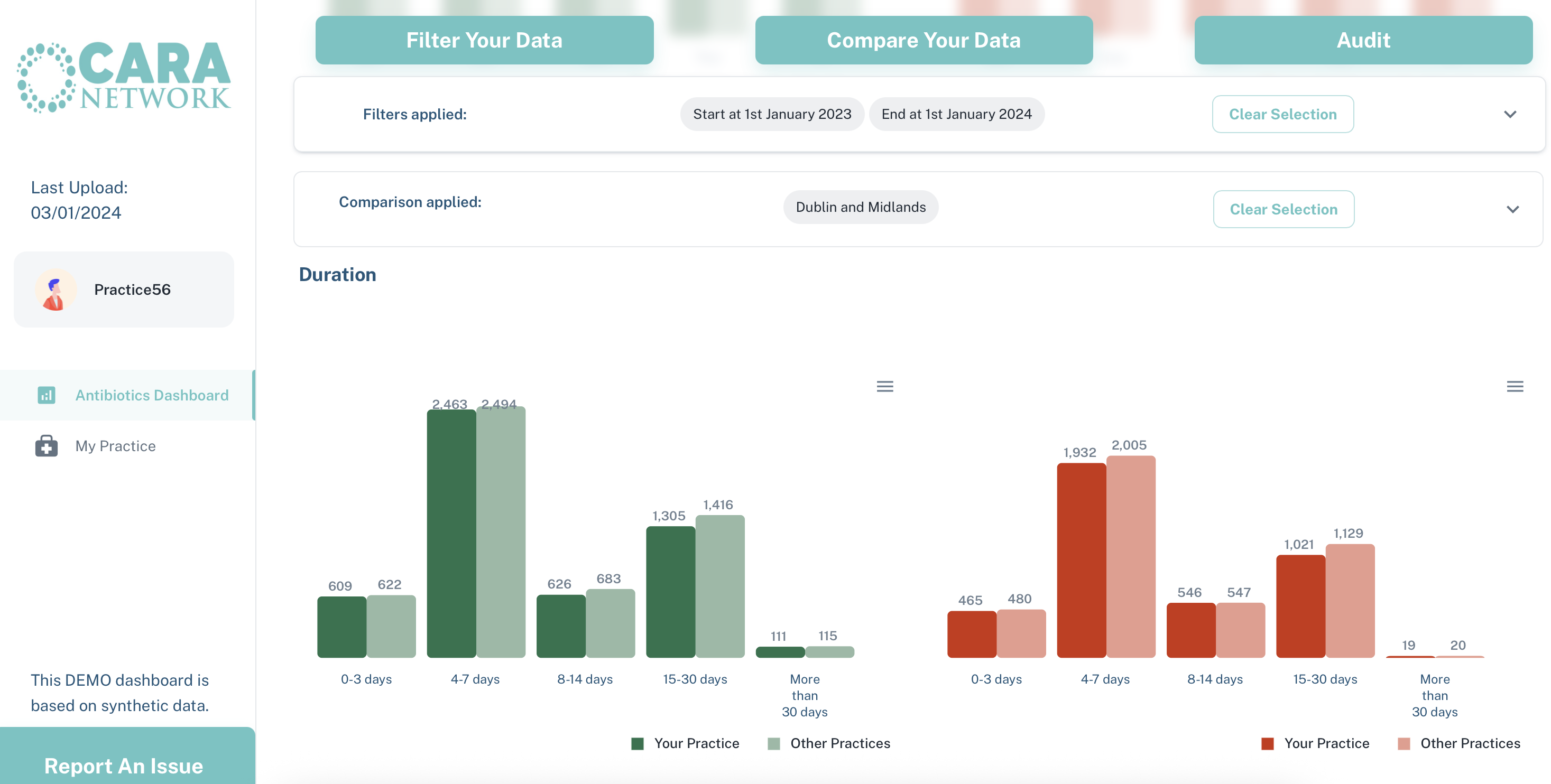 |

| **Link version** | **Action**: The dashboard needs to have a clear message and support / encourage users to take action. |
| --- | --- |
| **1** | 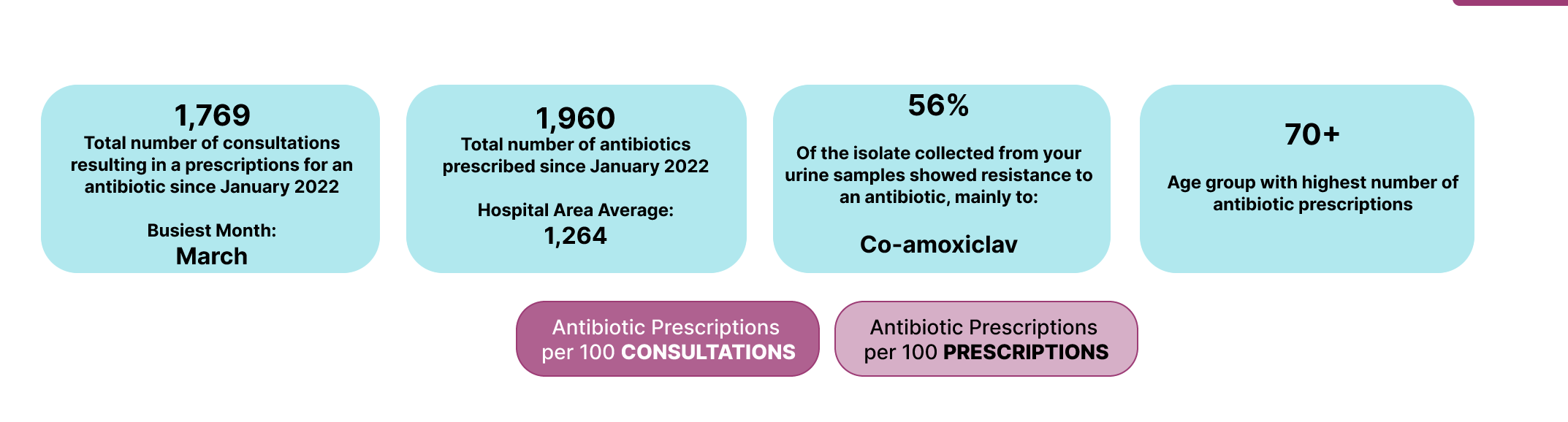 |
| **3** | 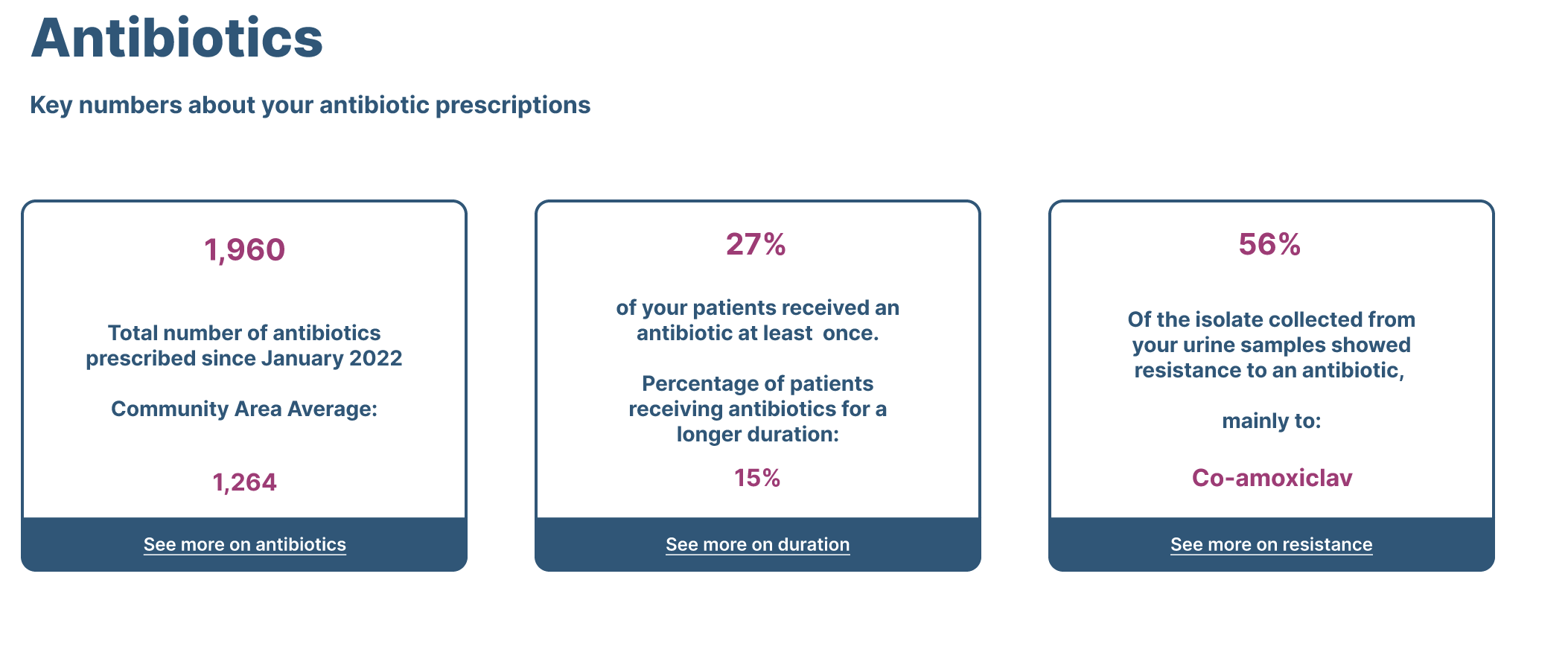 |
| **5** | 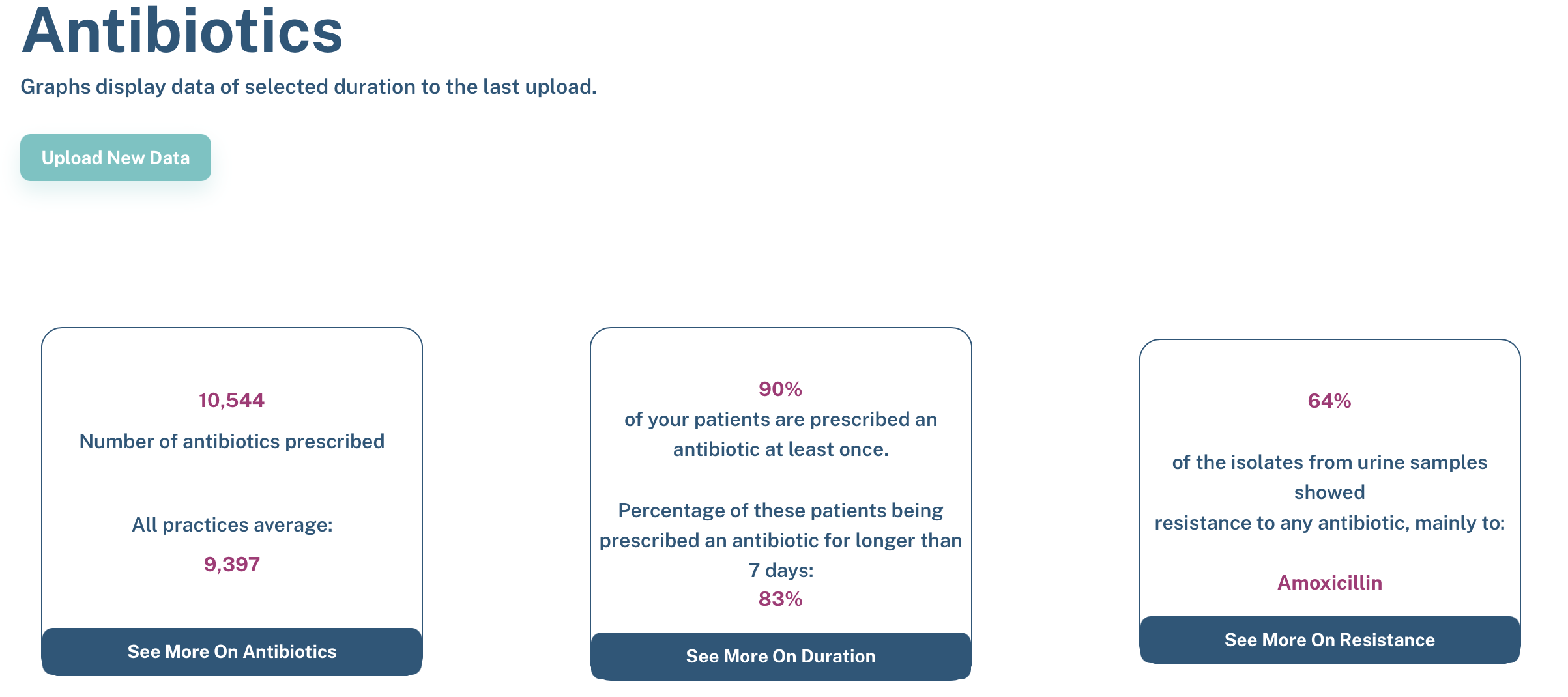 |

| **Link version** | **Engagement**: Users’ motivations and ongoing engagement need to be supported to be useful therefore users need to be kept in the loop. |
| --- | --- |
| **1** | 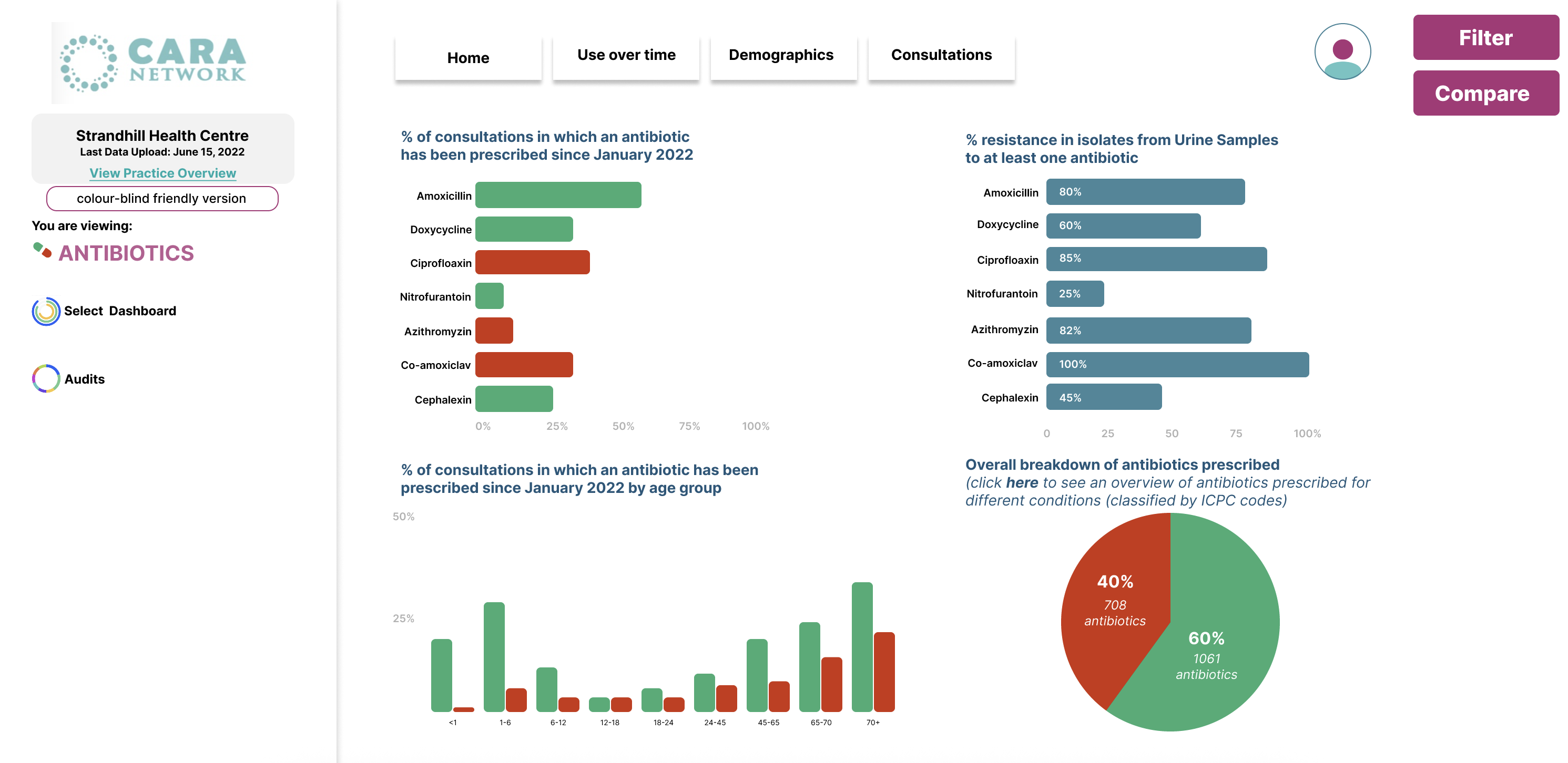 |
| **3** | 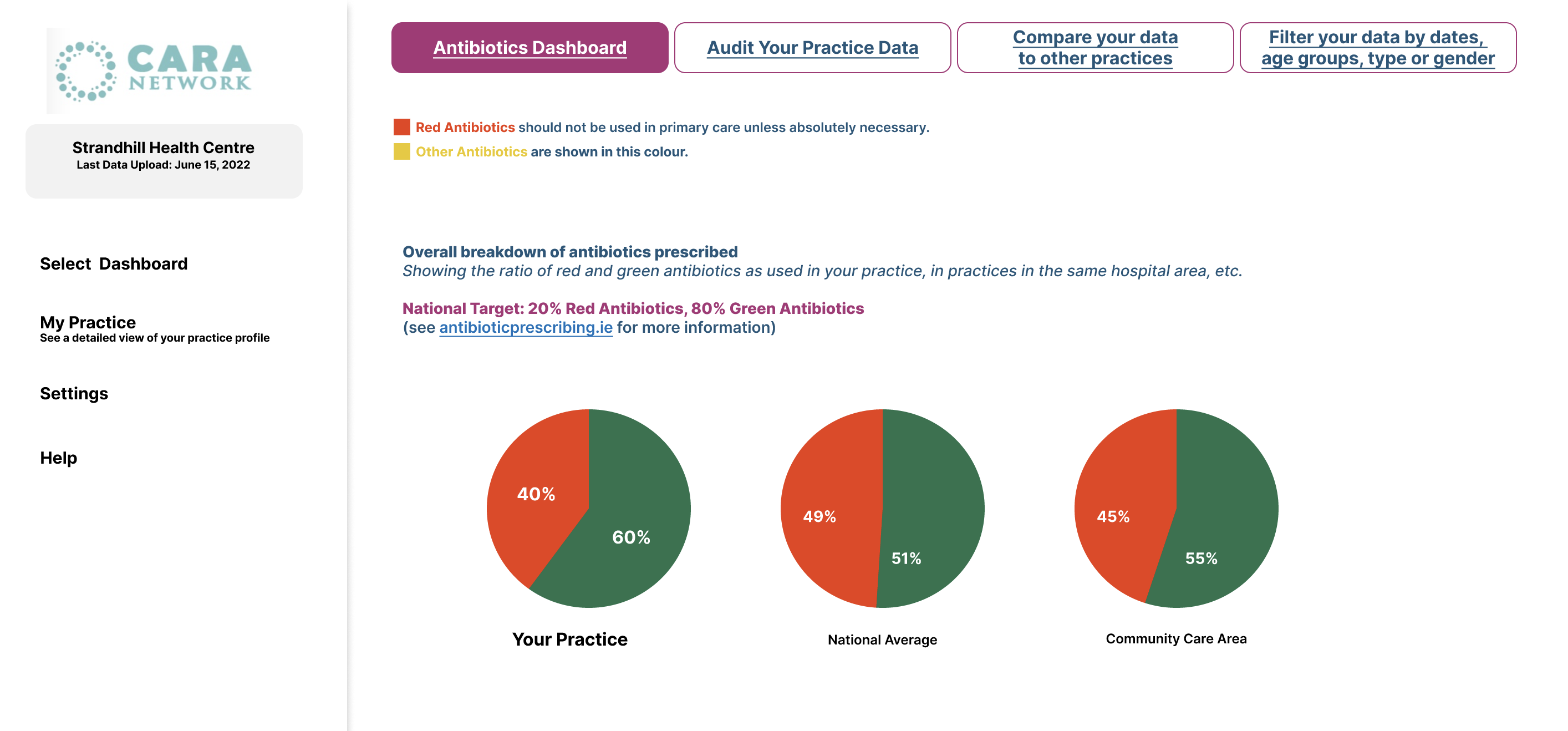 |
| **5** | 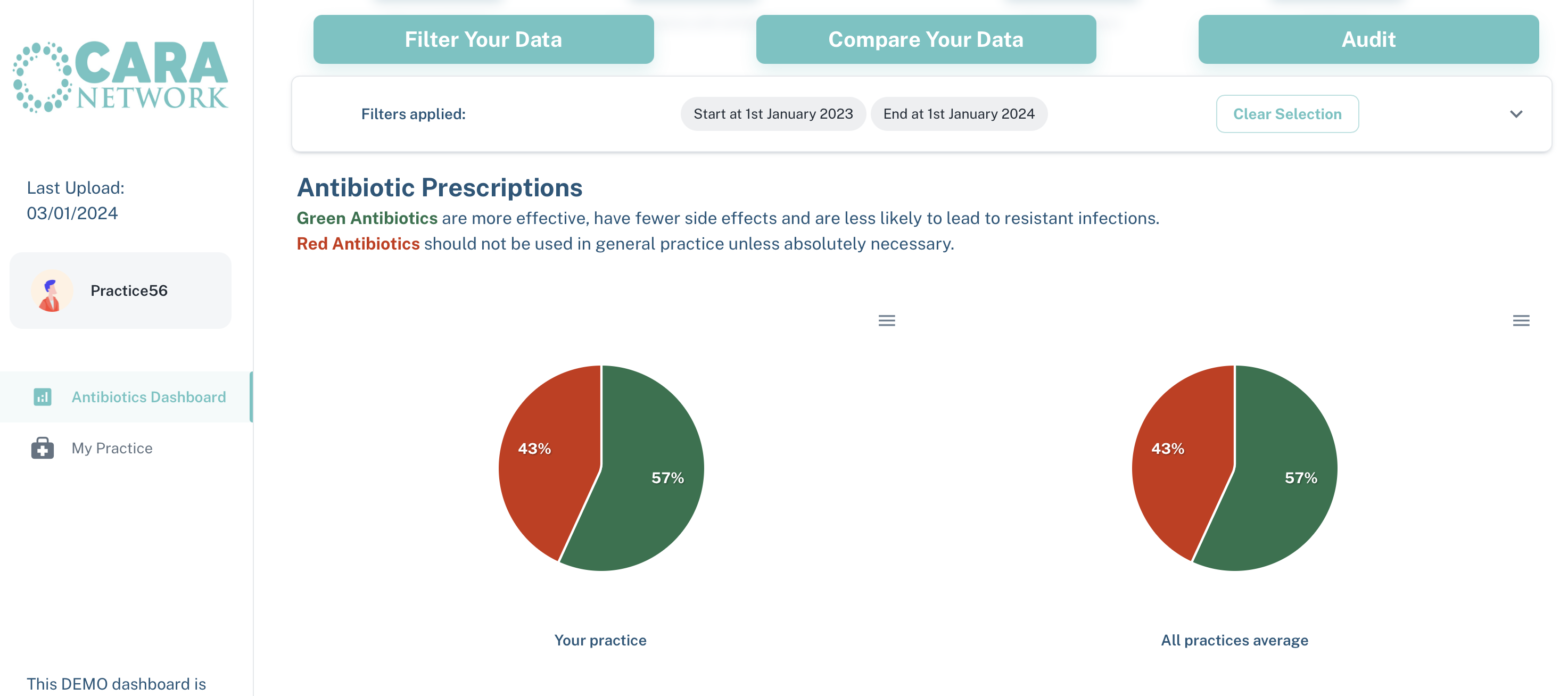 |

| **Link version** | **Engagement**: Users’ motivations and ongoing engagement need to be supported to be useful therefore users need to be kept in the loop. |
| --- | --- |
| **1** | 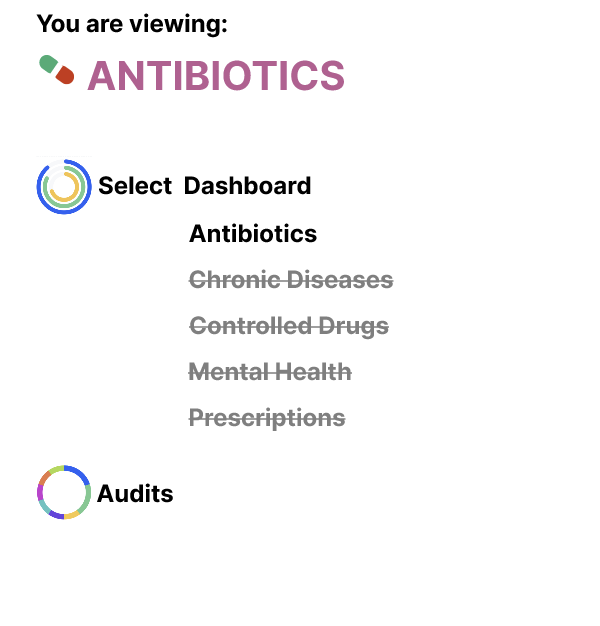 |
| **3** | 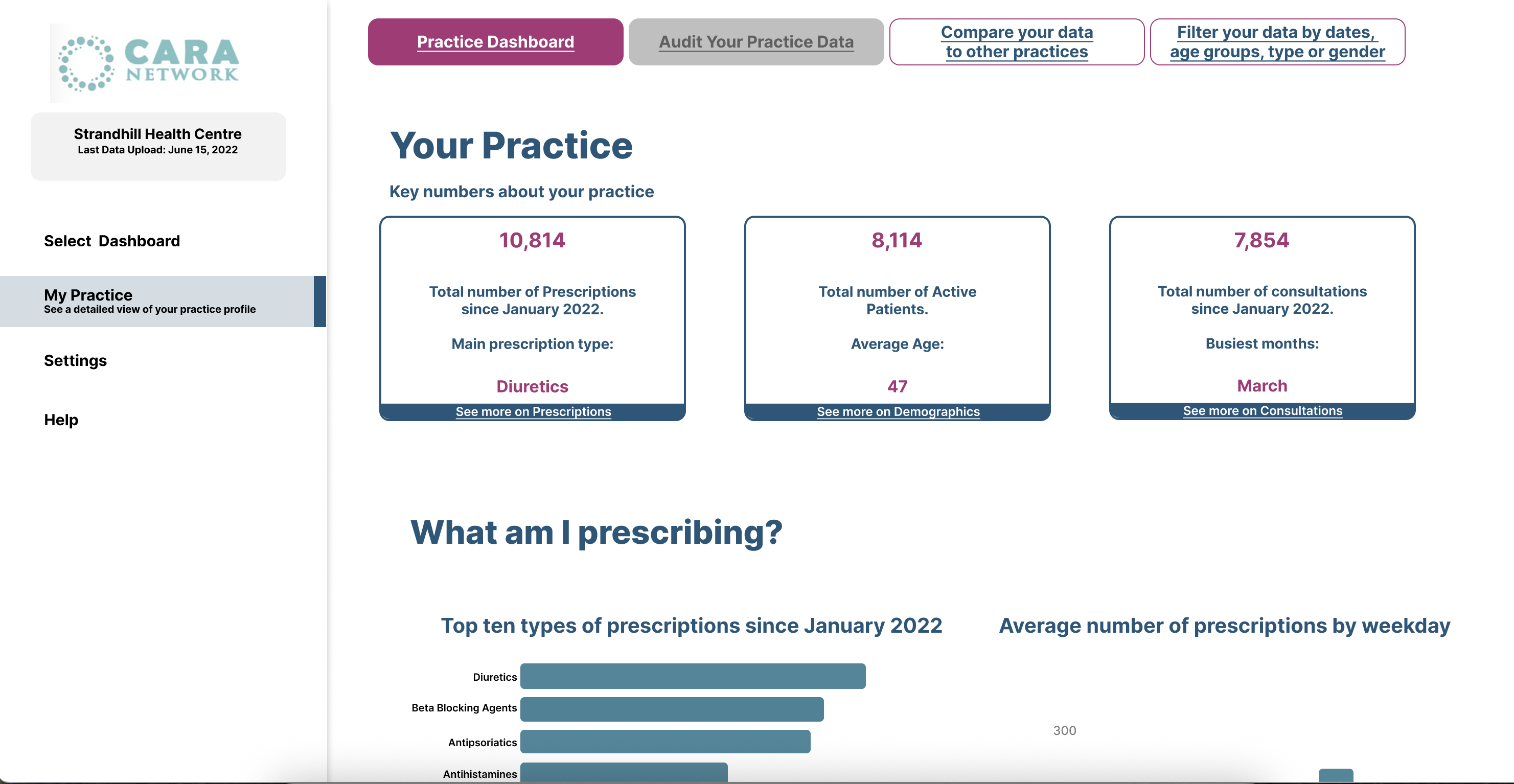 |
| **5** | 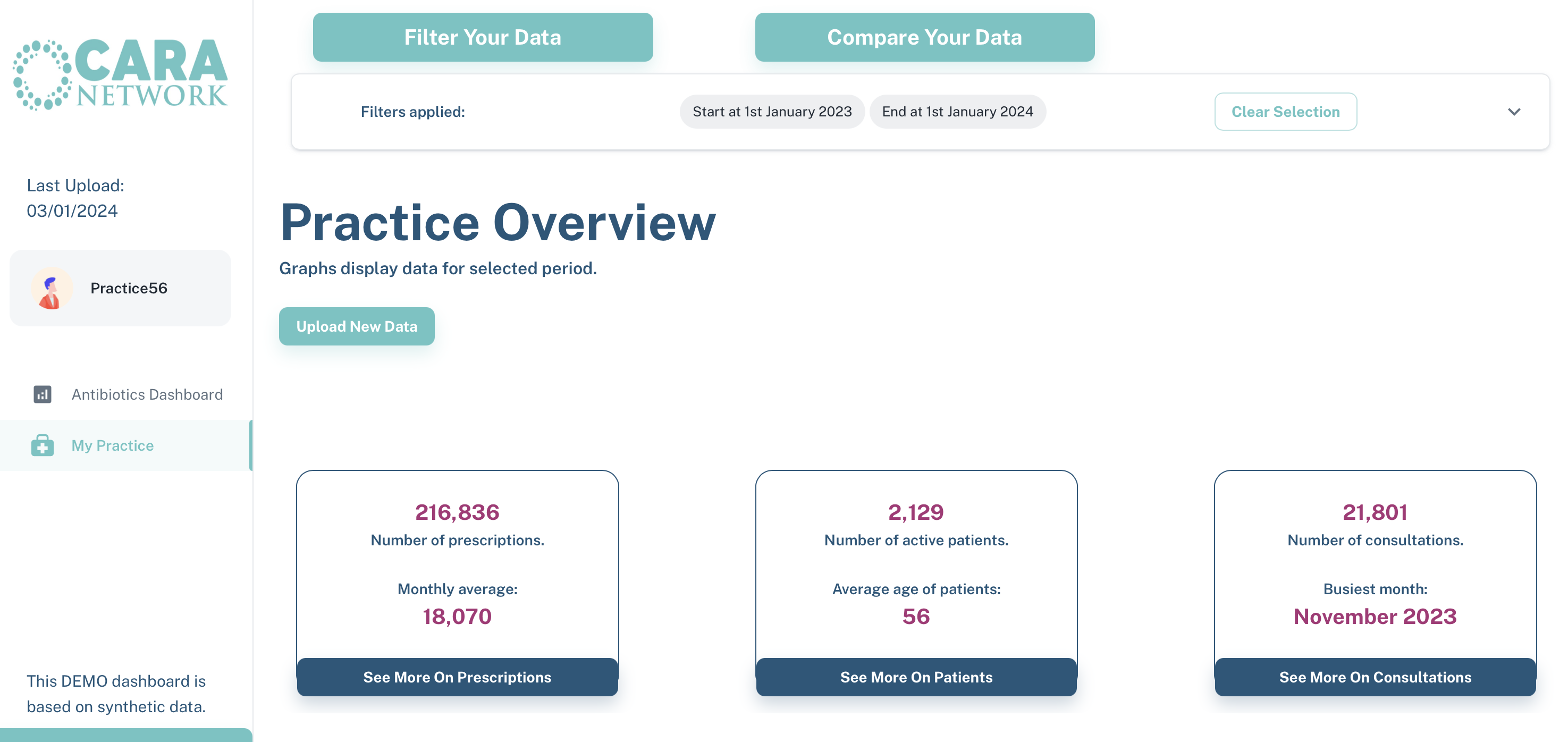 |

| **Link version** | **Ease of use:** Using a dashboard needs to be easy and efficient and cater for all levels of users to allow both data novices and experts to interact with the information. |
| --- | --- |
| **1 and 3** | 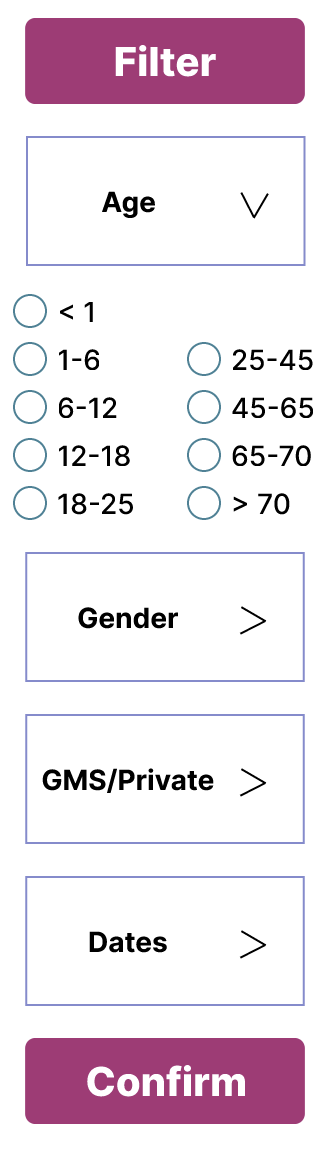 |
| **5** | 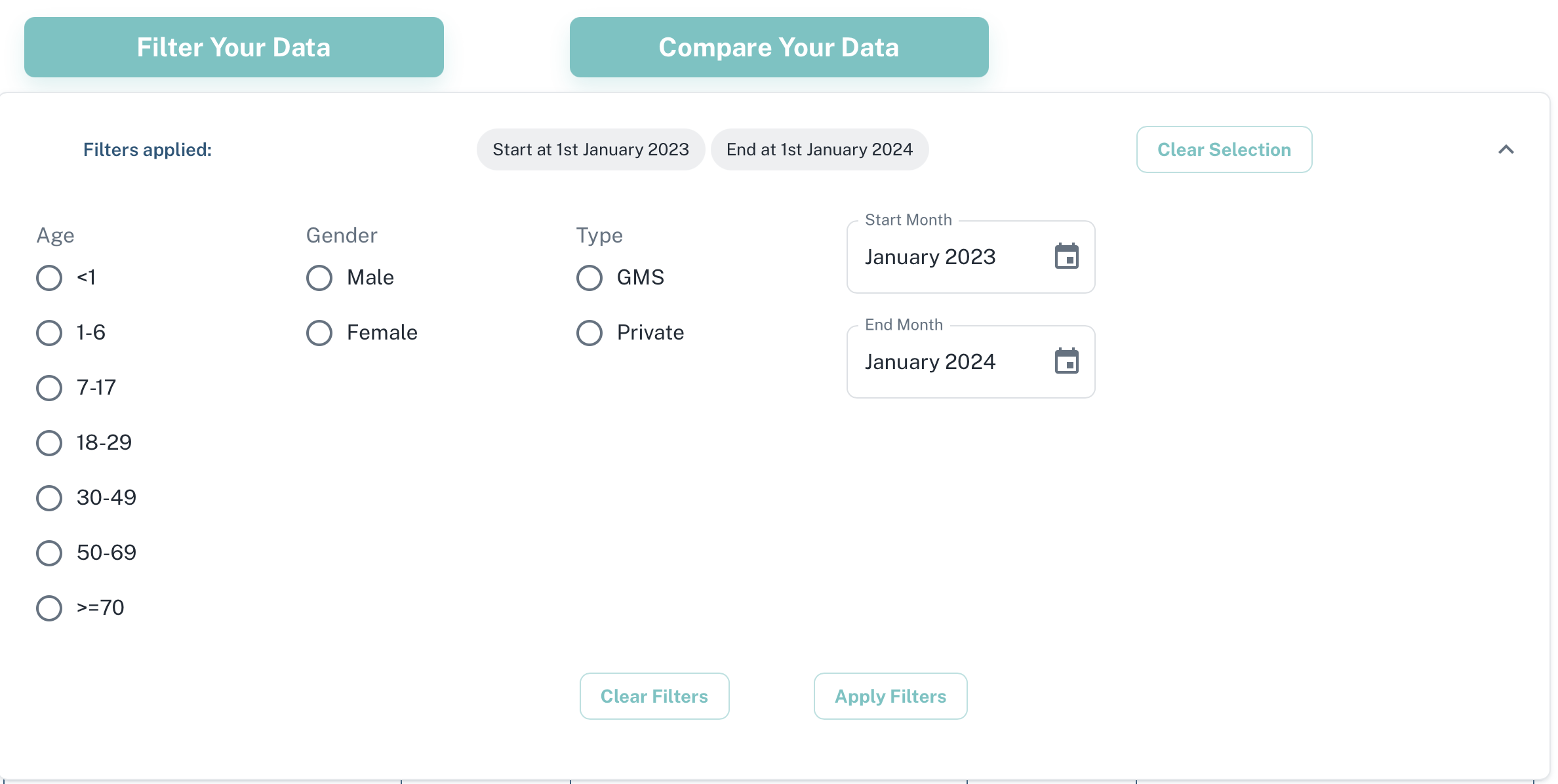 |

| **Link version** | **Ease of use:** Using a dashboard needs to be easy and efficient and cater for all levels of users to allow both data novices and experts to interact with the information. |
| --- | --- |
| **1** | 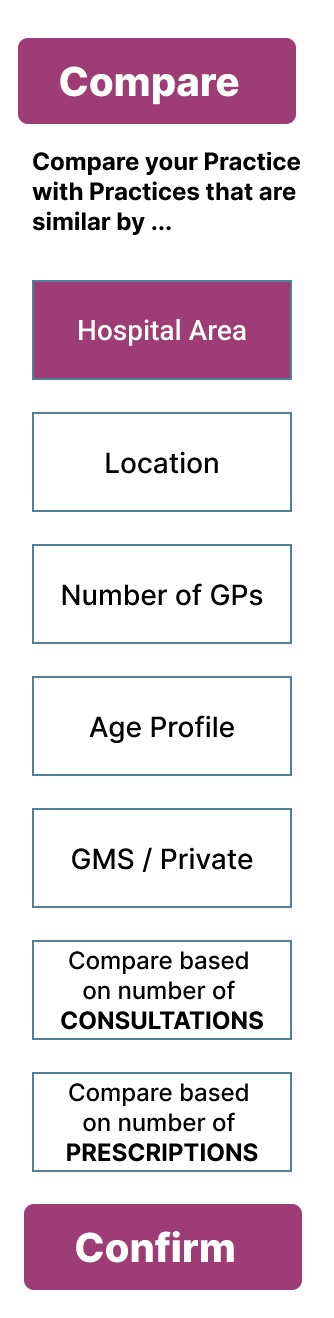 |
| **2** | 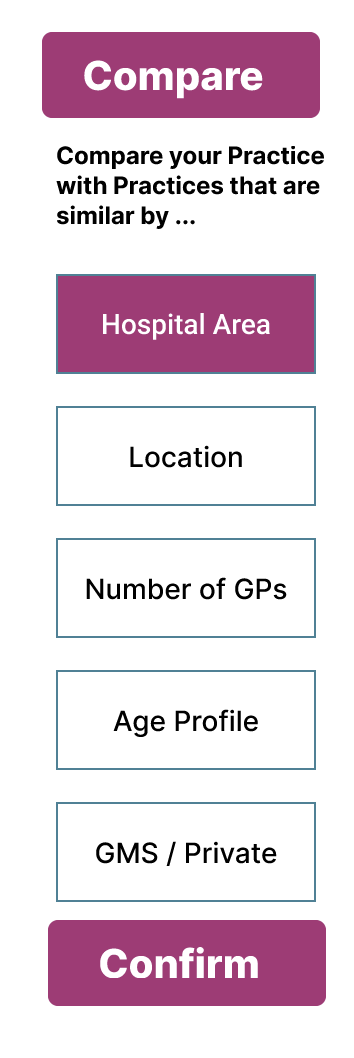 |
| **3** | 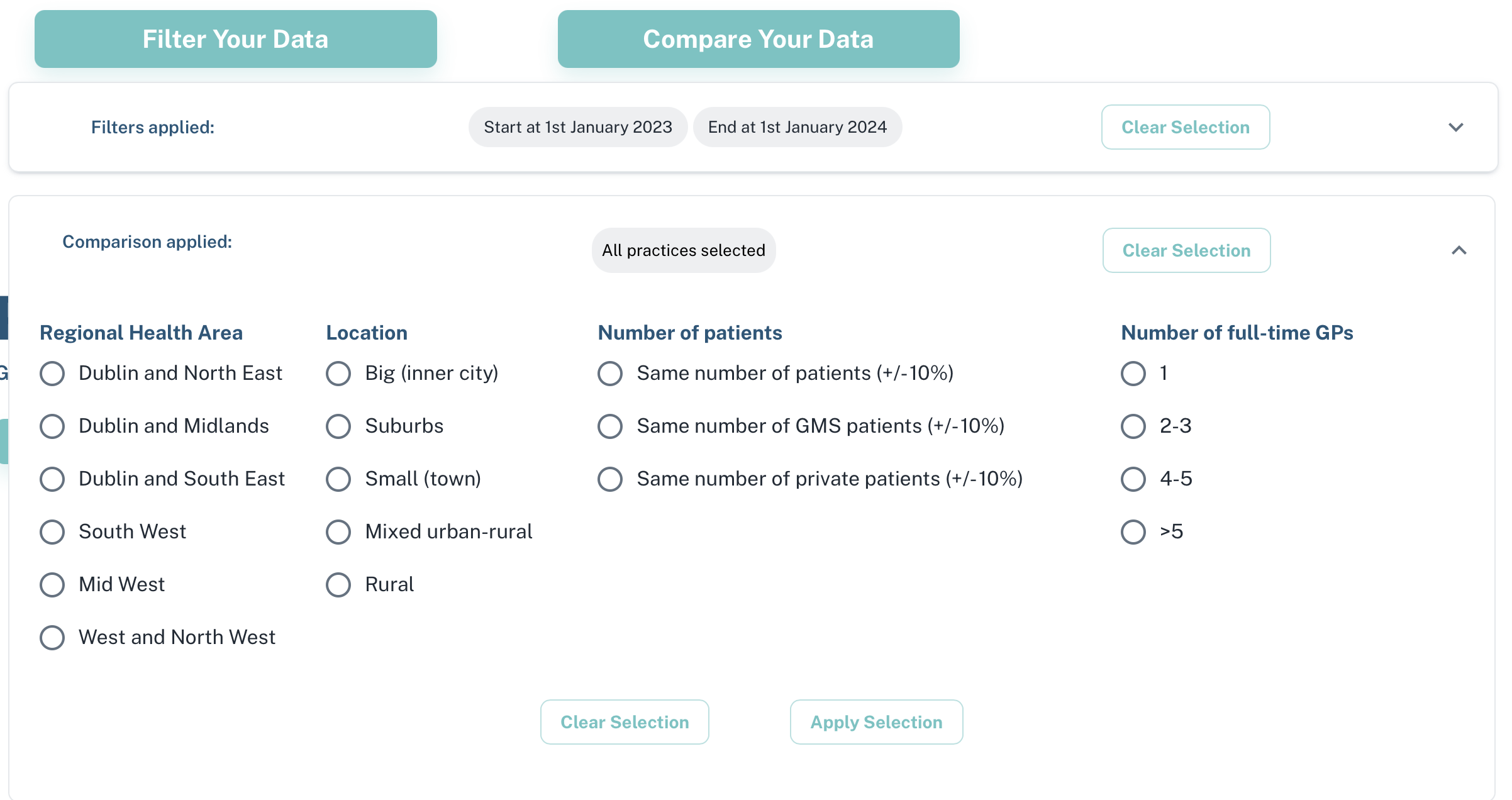 |

# Supplementary table 3. The main results of [Interviews 2]: Themes, categories and points to discuss by CARA team

**CARA dashboard prototype v.01:** [**https://www.figma.com/proto/6YgVhnHJGF2w7kMPj1Z241/Original-CARA-Prototype?scaling=scale-down-width&page-id=0%3A1&starting-point-node-id=2%3A2&node-id=2-2&hide-ui=1**](https://www.figma.com/proto/6YgVhnHJGF2w7kMPj1Z241/Original-CARA-Prototype?scaling=scale-down-width&page-id=0%3A1&starting-point-node-id=2%3A2&node-id=2-2&hide-ui=1)

| **Themes** | **Categories** | **Positive results** | **Points to discuss (CARA Team)** |
| --- | --- | --- | --- |
| **Audit** | Report | GPs found the report useful in terms of time and helpful in meeting presentations in practice (quick and efficient template) to show the trends and changes of their prescriptions.   - ’*It would be really nice because I just like co-workers in a big practice, there are seven or eight doctors, three or four nurses, and we have a meeting every Friday and morning, and like a clinical meeting of the half just before the patients come in, and different topics every week...And then you can reaudit this in a couple of months time. So yeah, I think it's really easy to understand it, and it's great, but yourself didn't have to troll through*’ (GP) | GPs mentioned too many options (graphs) to pick into the audit, and they could spend more time looking at the different charts and probably like to have it more directed towards.   - ’*They're almost too detailed, and I’m given maybe too many choices to pick from here, and I might spend more time looking at the different graphs that I've been trying to decide how they reduce this’* (GP)   Furthermore, GPs commented about the short time of the first chart in use over time (three months)   - ’ *Where aiming that might be good, use over time doesn't strike me so much because you'd expect it gets less in the summer and goes to peak here for some reason*’(GP) |
|  | Target or gold standard |  | GPs reflected the importance of including quality improvements by comparing their performance versus the gold standard or national target.   - '*The key thing you want is you want your own performance versus the gold standard versus your previous performance, Those are the three things I would like to see Anyway*' (GP)   Experts discussed possible targets or gold standards for the national level or focused on a specific drug.   - ’*Interestingly, there is a national target for the green and red antibiotics... The document I can send to you is the National Action Plan. There are two important targets there for you, and one is their overall. They want to reduce antibiotic prescribing in community settings by two per cent every year for the next four years*’ (Expert) |
| **Main antibiotics home page** | Summary (Key messages) | GPs found valuable the data summary before showing all charts.   - ’*I like this comparison to the average, so I think one of the challenges can be, what part how do I compare to everyone else? So this is useful*’ (GP) | GPs mentioned too many details in the summary and another additional summary they would like to see.   - '*Are you keeping up with your peers? Are you an outlier, and certainly didn't want to be? that's useful*' (GP)   Experts commented about summarising simply the antibiotic prescribing, comparing their prescription versus the health area or the national average (average of all practices). Furthermore, this section has a lot of information and details.   - ’ *I understand that there are practices doctors where are coming to find out about their antibiotic prescriptions, I may not know the detail of the particular use cases, and obviously, I am not a doctor, but coming in, I guess the first thing that strikes me is there's a lot of information…But immediately. I’m not clear what I can interact with*’ (Expert) |
|  | Guidelines |  | GPs discussed including a link to connect with national guidelines ([antibioticprescribing.ie](https://www.hse.ie/eng/services/list/2/gp/antibiotic-prescribing/)) and defining the green and red antibiotic categories.   - ’ *What are the Red ones? Again. Co-amoxiclav, azithromycin and ciprofloxacin*’ (GP) |
| **Design changes** | Location of icons |  | GPs and experts found difficulties in finding some icons such as practice overview, audit and going back to the main dashboard.   - ’ *Practice overview, where is that?’* (GP) |
|  | Change shades of Colours |  | - ’ *There are different shades...It's like you can go for the slightly dark or more contemporary green. There are slightly different shades. if you do that. And then if you made these they'd be just for nice big, square boxes, and we'll get them in for colours and again, maybe. Put them in the contemporary colours of further down’... (Expert)* |
|  | Interactive dashboard: setting up different areas |  | Experts suggested setting up the different areas on the outside and inside of the antibiotic dashboard.   - ’ *We have lots of different areas where we can interact with it and set things on down the left, across the top, over the right, in the middle, streamline and the main active areas, I would think, would be down here if it's something outside of antibiotics only (left side) and across here (top)*’ (*Expert*) |
|  | Filters and compare |  | Experts suggested organising both bottoms (filters and compare) and thinking about functionality and terminology of comparison.   - ’*Let's just say, for the like analysis, if you click on the analysis, you can compare with or do something else… Yes, the terminology just strikes me like I don't know what you mean. Compare like I'm comparing*’ (*Expert*) |
|  | Charts |  | Both GPs and experts suggested some changes to graphs, such as adding labels, explaining colours, clicking on the graph to select it, changing the header and others.   - Graph: % of consultations resulting in a prescription of red and green per weekday ’*And then on the weekdays you could probably compare. You could probably, combine those graphs. I don't know if you needed the two of them’* (GP) |
| **Practice Overview / My practice** |  | GPs found the practice overview helpful in terms of quality, practice management point of view and familiarity with practice activities.   - ’*This is certainly useful… it's interesting to us that looking at these sorts of factors are the types of things I would be interested in. So I think they're all very good. And again pretty clear*’... (GP) |  |
| **Use the dashboard** | Useful aspects | GPs discussed positive and valuable aspects of antibiotic stewardship, compared with other practices and could include in tutorial or training activities.   - *’ If you had identified antimicrobial stewardship as an issue for the practice, certainly it would be a useful sort of tool...we need the application to be in that bigger piece around the overall practice running… that would be very useful data in terms of GP workforce planning...especially if you had a national sort of a picture ...you could break it down into different problems that might be of relevance*’ (GP) |  |
|  | How to increase engagement and be more attractive to GPs |  | GPs mentioned that it takes a lot of work to get motivated GPs. They suggested including other dashboards, such as chronic diseases, to make this platform more attractive and increase engagement. Also, experts proposed including new analyses for future research.   - ’ *Have you a facility to look at delayed prescriptions (we will know the intention of the GP) versus immediate prescriptions?... Is it for the patient to take to the pharmacy immediately, or if they can? if it's a prescription that they say to the patients, I'm giving you the prescription now, but it's only kind of a backup prescription, and not to use it until unless you're symptoms. This improves in the next. You know hold is a whole. That you take it to the pharmacy immediately. You'd take it if you There was no improvement over this improvement in the next’* (Expert) |
| **Coding** |  |  | GPs were concerned about the accuracy of prescription and disease condition codes. They reflected that the platform should explain or define better these code systems (i.e. which code was used or included to merge the data).   - ’ *Is the prescribing stuff accurate that how? How is the actual sort of software program code the individualised items? Really, really well, and you can be sure that that drug is being prescribed... I don't know what ATC codes meaning*’...(GP) |
| **Protection data** |  |  | GPs discussed how the practice data is collected and protected on this platform.   - ' *And this data as this exported and anonymously... with the day to be used as well for research purposes...so long as it's anonymous data, I don't understand the way you're doing that*' (GP) |
| **Instruction and familiarity** |  |  | GPs reported needing help understanding the task in the audit section and being unfamiliar with this dashboard. They suggest strategies to improve these difficulties.   - ’ *I'm not familiar enough with your dashboard… the thing is the first time you do any task? It seems unfamiliar. It was, once, and or if there was another video or something like that, people's minds work differently… I would often just Youtube how to do something, and that's why I figured It's okay, and all the people want to see all the instructions, every single each other in the manual first*’ (GP)   Also, experts reflected that the dashboard's purpose needed to be clarified.   - ’ *I think the overall is the kind of purpose it's not clear now. I know I’m not a doctor, but I still think I should be stand-alone in terms of explaining. Yeah, it's purpose*’ (Expert) |
