# Supplementary material for: Action design research to develop an interactive dashboard to visualise and compare patient data from Irish general practice (CARA)
Source: BMJ Open. 2025 Sep 4;15(9):e086677. doi: 10.1136/bmjopen-2024-086677 (PMC12414190; doi:10.1136/bmjopen-2024-086677)
Supplement: Supplementary file 2 [file bmjopen-2024-086677.reviewer_comments.pdf]

PEER REVIEW HISTORY

BMJ Open publishes all reviews undertaken for accepted manuscripts. Reviewers are asked to complete a checklist review form and are provided with free text boxes to elaborate on their assessment. These free text comments are reproduced below.

ARTICLE DETAILS

Title (Provisional)

Action design research to develop an interactive dashboard to visualise and compare patient data from Irish general practice (CARA)

Authors

Vornhagen, Heike; Garzón-Orjuela, Nathaly; Stasiewicz, Katarzyna; Garcia Pereira, Agustin; Parveen, Sana; Porwol, Lukasz; Collins, Claire; Blake, Catherine; Vellinga, Akke

---

VERSION 1 - REVIEW

**Reviewer**	**1**
**Name**	**Ruddle, Roy**
**Affiliation**	**University of Leeds, School of Computing**
**Date**	**04-Jun-2024**
**COI**	**None**

The headline aim of this paper is stated as being to develop a dashboard for Irish GPs "to improve their understanding of their data". However, later on (last para of Intro) it becomes apparent that the paper's scope is actually much smaller, because it focusses just on a dashboard for antibiotic prescribing.

The Intro provides a brief review of previous work in healthcare dashboards, but the authors should explain how that work informed the design of their dashboard.

In an example of excellent practice, stakeholders were closely involved in the dashboard design. The involvement started with Interviews 1, which identified seven key challenges. The Results describes them in general terms, but much more detail is needed if readers are to be able to understand the difficulty of a dashboard addressing each challenge or to be able to generalise the research findings to other situations. For example, no sense is provided about "the potential volume of information available" – what is the scale and complexity of the prescribing data, data types, etc? And yes it makes sense that "easy generation of audit reports can save GPs time" but what are the requirements for doing that, how are such audits currently carried out and how long does that typically take?

The paper includes four figures showing various flowcharts, but only one figure showing what the dashboard actually looks like and that is for some rather simple audit output. The references and supplementary material contain a link to an external website for images of the first dashboard iteration (v.01), but the reader is left to themselves to try to ascertain how the dashboard meets what the paper claims about “thereby addressing challenges 2, 4, 5 and 7” and “allowed users to adjust the visualisations according to their need (challenge 1 and 4)”. It is obviously problematic to not include representative screenshots in the paper and/or supplementary material, because external websites can change and may also completely disappear.

That external website is interactive, but buggy. I filtered by Gender -> Male and was told that the website had applied two (not one) filters and they were Age: 70+ and Gender: Female! Then I selected Compare -> Age Profile and the dashboard said it was comparing “Similar Location: Hospital Area”!

The dashboard was developed in five iterations (see Fig 2). The supplementary material shows that Interviews 2 gathered some good and helpful-looking feedback at the end of the first iteration (e.g., “too many options” (audit), “too many details” (home page) and “explaining colours” (design changes)). However, the main body of the paper just says “The prototype was iteratively adjusted …” in a single sentence about the changes made in the dashboard itself for the other four iterations, with a little more detail provided about the audit changes.

In summary, I would say that this paper describes the process by which the dashboard was designed, rather than “describes the design and development of an interactive dashboard”. The lack of information about the scale and complexity of the prescribing data means that it is not possible to assess the magnitude of the challenges, and the lack of clear explanations about how the dashboard design addressed the details that underlie each challenge would impede readers’ ability to generalise the results.

Reviewer 2

Name Woodworth, Simon

Affiliation University College Cork National University of Ireland, Business Information Systems

Date 22-Jul-2024

COI None.

The study is interpretivist in nature and uses ADR. The approach is appropriate. It's not clear how many GPs were actually involved but clearly the number is small. Given the audience and the prevalence of statistical approaches in medical journals, you may wish to assert

more clearly the logical generalisability of your study. Siggelkow (2007) "Persuasion in Case Studies" is useful in this regard.

**Reviewer**	**3**
**Name**	**Schmiemann, Guido**
**Affiliation**	**Universität Bremen, Health Services Research**
**Date**	**21-Apr-2025**
**COI**	**I have no competing interests**

Thank you for the opportunity to review this interesting paper on the development of a dashboard for Irish GPs.

I have no concerns or major comments regarding the publication of this well written article.

Key aspects:

Background:

As the background to the development is mainly the situation in Irish GPs and the need for annual audits, I would suggest adding some information on this background.

Is there a system in place to give feedback to Irish GPs on their prescribing or performance data?

What have been the topics of clinical audits to date? Are these known in advance and do practices adapt their documentation to the information required?

Data analysis:

Why were different methods used to code the interviews (1 and 2)? Was there any checking/discussion after coding interview 1? What is meant by interviews (2) being checked by a second researcher? If not, how might this have influenced the results?

According to the COREQ checklist, data saturation was not controlled for - how might this have affected the results?

VERSION 1 - AUTHOR RESPONSE

#	Comments	Response

1	**Reviewer #1:**<br>The headline aim of this paper is stated as being to develop a dashboard for Irish GPs "to improve their understanding of their data". However, later on (last para of Intro) it becomes apparent that the paper's scope is actually much smaller, because it focusses just on a dashboard for antibiotic prescribing.<br>The Intro provides a brief review of previous work in healthcare dashboards, but the authors should explain how that work informed the design of their dashboard<br>In an example of excellent practice, stakeholders were closely involved in the dashboard design. The involvement started with Interviews 1, which identified seven key challenges. The Results describes them in general terms, but much more detail is needed if readers are to be able to understand the difficulty of a dashboard addressing each challenge or to be able to generalise the research findings to other situations. For example, no sense is provided about "the potential volume of information available" – what is the scale and complexity of the prescribing data, data types, etc? And yes it makes sense that "easy generation of audit reports can save GPs time" but what are the requirements for doing that, how are such audits currently carried out and how long does that typically take?	We thank the reviewer for their constructive comments and suggestions to improve our manuscript.<br><br>We agree that the mention of the antibiotic dashboard context in the 'Introduction' section may have caused confusion for the reader, particularly given that the primary aim of this paper is to describe the design and development process of the CARA dashboards. To address this, we revised the final part of the 'Introduction' section to clarify that the initial exemplar dashboard focused specifically on antibiotic prescribing and a practice overview (overview of practice demographics).<br>In addition, we removed the broader antibiotic context and incorporated information aligned with the reviewer's recommendations. These changes are reflected on pages 2 and 3, which now read:<br><br>"*Challenges arise across many areas, including data integration, scope of use and data complexity, which includes heterogeneous variables, multiple sources, missing and incomplete data and statistical rigour.*[1 2] *However, healthcare encounters additional barriers, due to stringent privacy regulations, high cost of clinical errors and the necessity for interpretations to align with established medical protocols.*[3 4] *Unlike other fields where exploratory analysis is encouraged, healthcare decisions require high precision, limiting the flexibility of visual analytics and thereby slowing their adoption.*[3 4]<br>*……dashboards are typically launched for a single purpose, such as research, exploration, analysis or business. Most dashboards are designed 'for' users rather than 'with' them, offering limited or no user interaction or engagement.* [5-7] *Once set up, dashboards require ongoing input from the developers to maintain their relevance,* [5] *which must align with the end users' needs. Developers should apply iterative performance evaluations during the development and implementation phase to encourage uptake and use.*[8] *Researchers and developers must ensure the dashboard remains effective and relevant in terms of both health outcomes and user engagement.*"

2	The paper includes four figures showing various flowcharts, but only one figure showing what the dashboard actually looks like and that is for some rather simple audit output. The references and supplementary material contain a link to an external website for images of the first dashboard iteration (v.01), but the reader is left to themselves to try to ascertain how the dashboard meets what the paper claims about "thereby addressing challenges 2, 4, 5 and 7" and "allowed users to adjust the visualisations according to their need (challenge 1 and 4)". It is obviously problematic to not include representative screenshots in the paper and/or supplementary material, because external websites can change and may also completely disappear.	We added supplementary material (table 3) to show screenshots illustrating how specific challenges were addressed during the design process of the CARA dashboard. A sentence had also been added on page 6, which now read:<br><br>"*Supplementary table 3 provides visual evidence (screenshots) illustrating how specific challenges were identified and addressed during the design process of the CARA dashboard*"
3	That external website is interactive, but buggy. I filtered by Gender -> Male and was told that the website had applied two (not one) filters and they were Age: 70+ and Gender: Female! Then I selected Compare -> Age Profile and the dashboard said it was comparing "Similar Location: Hospital Area"!	The first link (Reference 33) directed to an early prototype created in Figma. This was not an interactive website, but rather a static design mock-up, which explains its limited functionality. The second link (Reference 34) originally pointed to the final version of the CARA dashboard. However, since the previous URL (http://vmcara-vm2.datascienceinstitute.ie/dashboard/app) is no longer active, we have replaced it with a new link to the DEMO version of the dashboard (https://demo.caranetwork.ie).

4	The dashboard was developed in five iterations (see Fig 2). The supplementary material shows that Interviews 2 gathered some good and helpful-looking feedback at the end of the first iteration (e.g., “too many options” (audit), “too many details” (home page) and “explaining colours” (design changes)). However, the main body of the paper just says “The prototype was iteratively adjusted …” in a single sentence about the changes made in the dashboard itself for the other four iterations, with a little more detail provided about the audit changes. In summary, I would say that this paper describes the process by which the dashboard was designed, rather than “describes the design and development of an interactive dashboard”. The lack of information about the scale and complexity of the prescribing data means that it is not possible to assess the magnitude of the challenges, and the lack of clear explanations about how the dashboard design addressed the details that underlie each challenge would impede readers' ability to generalise the results.	Thank you for your comments, we added supplementary materials (see response 2) which show the changes made and includes screenshots and three different version of the dashboard, allowing the reader to identify the adjustments and the challenges addressed.
5	**Reviewer #2:**<br>The study is interpretivist in nature and uses ADR. The approach is appropriate. It's not clear how many GPs were actually involved but clearly the number is small. Given the audience and the prevalence of statistical approaches in medical journals, you may wish to assert more clearly the logical generalisability of your study. Siggelkow (2007) "Persuasion in Case Studies" is useful in this regard.	We appreciate your comment and suggestion to improve our manuscript.<br>We clarified in the ‘Results' section how many GPs were included, page 5, which now reads:<br><br>“*Sixteen interviews were conducted in total: six in [Interviews 1] with GPs (‘Problem formulation') and ten in [Interviews 2], which included five GPs who had already participated in the [Interviews 1], two newly recruited GPs and three experts (‘Building, intervention and evaluation')*”<br><br>In the ‘Discussion' section (page 7), we have addressed the logical generalisability of our study and included a

		citation to Siggelkow (2007), as you suggested. The revised text now reads:<br>"*This study followed an ADR approach, which emphasises iterative problem-solving through cycles of artifact construction, intervention and evaluation.* [23] *Sixteen interviews were conducted to inform the iterative process; however, the goal was not to achieve data saturation, as would be expected in traditional qualitative methodologies such as grounded theory. Instead, the aim was to support the ongoing refinement of the dashboard through continuous stakeholder engagement. Accordingly, the sample size was determined by the pragmatic requirements of the design process rather than by the pursuit of thematic saturation. Given the study's interpretivist orientation, generalisability is analytical rather than statistical. Case-based and design-oriented research can yield theoretical insights that are transferable beyond the immediate empirical context.*[32] *In this case, the lessons learned about co-designing and implementing dashboards in general practice may have relevance for similar initiatives in other healthcare settings*"
6	**Reviewer #3:**<br>Thank you for the opportunity to review this interesting paper on the development of a dashboard for Irish GPs.<br>I have no concerns or major comments regarding the publication of this well written article.	We thank the reviewer for their approval of the manuscript.
7	Background:<br>As the background to the development is mainly the situation in Irish GPs and the need for annual audits, I would suggest adding some information on this background. Is there a system in place to give feedback to Irish GPs on their prescribing or performance data? What have been the topics of clinical audits to date? Are these known in advance and do practices adapt their documentation to the information required?	Thank you for your comment. We appreciate your suggestion. We added information in the 'Introduction' section about initiatives in Ireland related to feedback mechanisms, page 3, which now reads:<br><br>"*The government has implemented some initiatives such as Summary Care Records (SCRs) and ePrescribing, which aim to improve interoperability. However these efforts have not resulted in the integration of data visualisation tools for general practice.*[21] *Moreover, data integration and visualisation remain underdeveloped in key areas, particularly in prescribing feedback mechanisms. The Health Service Executive (HSE) Primary Care Reimbursement Service has introduced quarterly antibiotic prescribing feedback for patients with a medical card.* [22 23] *However, this feedback excludes information on private patients is in pdf format and has been reported as complex, lacking interactive features.*[24] *Although these initiatives aim to*

		integrate health services, they are designed for specific purposes and rarely seek input from GPs during development or implementation. No initiative facilitates comparisons with other practices or national targets, making it difficult to benchmark and assess GPs' prescribing and care delivery"
8	Data analysis:<br>Why were different methods used to code the interviews (1 and 2)? Was there any checking/discussion after coding interview 1? What is meant by interviews (2) being checked by a second researcher? If not, how might this have influenced the results?<br>According to the COREQ checklist, data saturation was not controlled for - how might this have affected the results?	The methods were different because in interview 1 these were informed by the research questions and focused on understanding what type of information needs GPs have. The second interview focused more on the different sections of the dashboard and how participants understand these (user experience review). In the second interview, having a second researcher check the coding helped to identify and group themes and categories relevant to the user experience. The first interview was more exploratory in nature and facilitated the identification of areas to visualise whereas the second interview was concerned with categorising interview data.<br><br>Regarding data saturation (see response 5). We added a statement in the 'Discussion' section (page 7) to clarify this point. Since the dashboard is being developed using the Action Design Research (ADR) approach, the aim is not to achieve data saturation, as would be the case in grounded theory or other purely qualitative approaches. Rather, ADR focuses on iterative problem-solving and continuous refinement of the dashboard, which is why data saturation is not a central objective in this context.

VERSION 2 - REVIEW

**Reviewer**	**1**
**Name**	**Ruddle, Roy**
**Affiliation**	**University of Leeds, School of Computing**
**Date**	**31-Jul-2025**
**COI**	

Thank you to the authors for their revised manuscript and response to reviewers.

I raised six main issues in my review of the original manuscript. The authors' response groups together the first three issues, and the first is addressed by the revisions which now state that the focus is just on antibiotic prescribing and practice demographics. However, neither #2 or #3 have been adequately addressed.

Issue 2 (“*The Intro provides a brief review of previous work in healthcare dashboards, but the authors should explain how that work informed the design of their dashboard.*”) is only discussed in the most general terms, with the addition of comments about the need for iterative development and to involve users in the design. Those points are long-established and apply to any interactive system.

My first-round review noted the seven key challenges were identified during stakeholder interviews and that the Results describe the challenges in general terms. However, the issue (#3) was that:

> *… much more detail is needed if readers are to be able to understand the difficulty of a dashboard addressing each challenge or to be able to generalise the research findings to other situations. For example, no sense is provided about “the potential volume of information available” – what is the scale and complexity of the prescribing data, data types, etc? And yes it makes sense that “easy generation of audit reports can save GPs time” but what are the requirements for doing that, how are such audits currently carried out and how long does that typically take?*

That issue is not addressed in the authors’ response to reviewers and none of the changes to the manuscript enlighten readers about the difficulty of addressing each challenge.

Issue 4 commented that there was *“only one figure showing what the dashboard actually looks like”* (a fuzzy screen dump of some rather simple audit output) and that:

> *… the reader is left to themselves to try to ascertain how the dashboard meets what the paper claims about “thereby addressing challenges 2, 4, 5 and 7” and “allowed users to adjust the visualisations according to their need (challenge 1 and 4)”.*

The authors’ response has been to add one sentence to the manuscript (“Supplementary table 3 provides visual evidence …”), which points the reader to 16 pages of screenshots that have been added to the supplementary material. According to that supplementary table’s headings, there are screenshots for each challenge. However, readers are left to work out for themselves how users’ requirements were addressed – there is no actual explanation whatsoever.

In response to Issue 5 (the buggy, external website) the authors’ have changed the references to point to different versions of the dashboard.

Issue 6 noted that *“The dashboard was developed in five iterations (see Fig 2)”* but that four of the five iterations were covered in a single sentence about the prototype being iteratively adjusted. The authors’ response just points to the new supplementary table 3 (see above), which is not adequate because there is no explanation and readers are left to work things out for themselves.

In addition, that Fig 2 refers to dashboard versions 0.1, 0.2, 0.3, 0.4 and 1.0. However, 0.2, 0.3 and 0.4 are not mentioned at all in the body of the manuscript’s text. Further confusing matters, the supplementary table 3 refers to an original prototype (is that v0.1?), final prototype (I assume that is v1.0) and a DEMO dashboard which seems to be subsequent work.

Reviewer 2

Name Woodworth, Simon

Affiliation University College Cork National University of Ireland, Business Information Systems

Date 12-Jun-2025

COI

I have answered "no" to 4 review questions but these are very mild no's. I am recommending some minor revisions as follows.

1. Be very clear about your objective at the end of your introduction.

2. Can you state the outcomes a little more clearly? What is novel here?

3. Ad per (2), state your results clearly. I know they are in the supplemental material but perhaps a little more in the main body?

4. I think you need to tackle the study limitations a bit more. This is of course a qualitative study so sample size is not at issue. Nevertheless 16 respondents is a little small, I would have hoped for twice this amount. That said, your citation of Siggelkow strengthens your case for logical generalisability.

5. You might be more specific about a follow on study. A survey perhaps?

All that said, I like this paper and my comments and responses should be taken as suggestions.

**Reviewer**	**3**
**Name**	**Schmiemann, Guido**
**Affiliation**	**Universität Bremen, Health Services Research**
**Date**	**01-Jun-2025**
**COI**	

Thank you for addressing the reviewer's comments so adequately and thoroughly.I have no further remarks

VERSION 2 - AUTHOR RESPONSE

#	Comments	Response
5	**Reviewer 1**:<br>Thank you to the authors for their revised manuscript and response to reviewers.<br>I raised six main issues in my review of the original manuscript. The authors' response groups together the first three issues, and the first is addressed by the revisions which now state that the focus is just on antibiotic prescribing and	We thank the reviewer for the comments and suggestions to improve our manuscript.<br><br>We revised the final paragraph of the Introduction to integrate how prior works informed the design of the CARA dashboard. This change is reflected on page 4, which now reads:<br><br>"*Internationally, similar dashboards focused on antibiotic prescribing that include user involvement, have been developed in Canada and the UK.* [26 27] *However, these dashboards do not describe the process*

	practice demographics. However, neither #2 or #3 have been adequately addressed. Issue 2 ("The Intro provides a brief review of previous work in healthcare dashboards, but the authors should explain how that work informed the design of their dashboard.") is only discussed in the most general terms, with the addition of comments about the need for iterative development and to involve users in the design. Those points are long-established and apply to any interactive system.	*of user involvement in the system design, development and testing or use a methodological framework for developing the dashboard. A recent scoping review of dashboards in health care identified major shortcomings in how dashboards are typically developed.* [28] *Of the 118 dashboards reviewed, only 50% reported involving end users in the development process and even fewer (22%) included usability testing.* [28] *Informed by these shortcomings, as well as by the fragmented data environment and limited visualisation tools with general practice data, CARA adopted a structured methodological framework with continuous GP participation, co-design principles and iterative testing. The aim….*"
6	My first-round review noted the seven key challenges were identified during stakeholder interviews and that the Results describe the challenges in general terms. However, the issue (#3) was that: ... much more detail is needed if readers are to be able to understand the difficulty of a dashboard addressing each challenge or to be able to generalise the research findings to other situations. For example, no sense is provided about "the potential volume of information available" – what is the scale and complexity of the prescribing data, data types, etc? And yes it makes sense that "easy generation of audit reports can save GPs time" but what are the requirements for doing that, how are such audits currently carried out and how long does that typically take?<br>That issue is not addressed in the authors' response to reviewers and none of the changes to the manuscript enlighten readers about the difficulty of addressing each challenge.	We added a table to expand the Results section to provide more specific information on each challenge, including the reason or requirement of these challenges. This table 1 is included on pages 7 and 8.
7	Issue 4 commented that there was "only one figure showing	We added a table to expand the Results section to provide more specific information on each challenge,

	what the dashboard actually looks like" (a fuzzy screen dump of some rather simple audit output) and that: ... the reader is left to themselves to try to ascertain how the dashboard meets what the paper claims about "thereby addressing challenges 2, 4, 5 and 7" and "allowed users to adjust the visualisations according to their need (challenge 1 and 4)".<br>The authors' response has been to add one sentence to the manuscript ("Supplementary table 3 provides visual evidence ..."), which points the reader to 16 pages of screenshots that have been added to the supplementary material.<br>According to that supplementary table's headings, there are screenshots for each challenge. However, readers are left to work out for themselves how users' requirements were addressed – there is no actual explanation whatsoever.	including how we addressed these challenges "*addressed by*". This table 1 is included on pages 7 and 8.
8	In response to Issue 5 (the buggy, external website) the authors' have changed the references to point to different versions of the dashboard.	We organised the external websites and included the link to the prototype versions (see references 34 to 37).
9	Issue 6 noted that "The dashboard was developed in five iterations (see Fig 2)" but that four of the five iterations were covered in a single sentence about the prototype being iteratively adjusted. The authors' response just points to the new supplementary table 3 (see above), which is not adequate because there is no explanation and readers are left to work things out for themselves.	We added a table to the Results section to provide more specific information on each challenge, including the main changes to the prototype made in response to each challenge. This table 1 is presented on pages 7 and 8.
10	In addition, that Fig 2 refers to dashboard versions 0.1, 0.2, 0.3, 0.4 and 1.0. However, 0.2, 0.3 and 0.4 are not mentioned at all	We added the four versions of CARA prototypes (table 1 and references 34 to 37) in the body of the text and extended the information on the DEMO version. This change is included on page 6, which now reads:

	in the body of the manuscript's text. Further confusing matters, the supplementary table 3 refers to an original prototype (is that v0.1?), final prototype (I assume that is v1.0) and a DEMO dashboard which seems to be subsequent work.	"*Figure 2 shows the design cycle process from the CARA dashboard prototype v0.1* [34-37] *to the CARA dashboard v1.0 (which was created specifically for the workshop and is no longer available. Instead a new demo version with synthetic practice data was created).* [38]"
11	**Reviewer 2**:<br>I have answered "no" to 4 review questions but these are very mild no's. I am recommending some minor revisions as follows.<br><br>1. Be very clear about your objective at the end of your introduction.	We thank the reviewer for the comments and suggestions to improve our manuscript.<br><br>We clarified the objective of this study. This change is reflected on page 4, which now reads:<br><br>"*The aim of this study was to design and develop interactive CARA dashboards to enable Irish GPs to visualise their patient data and compare their data with other practices*"
12	2. Can you state the outcomes a little more clearly? What is novel here?	We added the outcome and novelty of this study to the methods section . This change is included on page 4, which now reads:<br><br>"*The primary outcome of this ADR process was the development of a prototype CARA dashboard tailored to Irish GPs, enabling visualisation of their data, comparison with other practices and audit generation. A key feature of this work is its iterative, co-designed development, informed directly by end-users through interviews and workshop feedback. The ADR approach also facilitated the generation of transferrable design principles for dashboard development in general practice, representing a novel contribution to both dashboard design methodology and primary care informatics.*"
13	3. Ad per (2), state your results clearly. I know they are in the supplemental material but perhaps a little more in the main body?	We added a table 1 to provide more information to the results section and to summarise how specific challenges were addressed during the design process of the CARA dashboard.
14	4. I think you need to tackle the study limitations a bit more. This is of course a qualitative study so sample size is not at issue. Nevertheless 16 respondents is a little small, I would have hoped for twice this amount. That said, your citation of Siggelkow strengthens your case for logical generalisability.	We discussed the limitation in the discussion section. The changes are reflected on page 11, see editor comment (#3).

15	5. You might be more specific about a follow on study. A survey perhaps?<br><br>All that said, I like this paper and my comments and responses should be taken as suggestions.	The dashboard and infrastructure were launched in 2024. This paper has remained under review with the journal for over a year and a half. During this period, we conducted a feasibility study of the infrastructure and dashboards in real-world settings and a user experience study involving GPs and researchers using synthetic data (DEMO dashboard), which included a survey. These studies are currently in the process of being published. We have referred to this work at the end of the discussion section, as reflected on page 11, which now reads:<br><br>"*However, the CARA dashboards and infrastructure were launched in early 2024* [25]. *Since, several actions have been undertaken to generalise the findings. These include exploring the technical feasibility of the CARA infrastructure and dashboards in real-world settings and conducting a user experience study with GPs and researchers using synthetic data (DEMO dashboard).* [38]"
16	Reviewer: 3<br>Thank you for addressing the reviewer's comments so adequately and thoroughly. I have no further remarks	We sincerely thank the reviewer for approving the manuscript. We appreciate their time and thoughtful review.
